# Shallow permeability structure and gas flow in hydrothermally altered soils at the Rotokawa Geothermal Field, New Zealand

**DOI:** 10.1007/s00445-026-02002-7

**Published:** 2026-06-23

**Authors:** Roberto Davoli, Giancarlo Tamburello, Tullio Ricci, Cristian Montanaro, Riccardo Civico, Shane J. Cronin, Farrell Siega, Carlo Cardellini, Thomas J. Jones, Bettina Scheu

**Affiliations:** 1 Earth and Environmental Sciences, Ludwig-Maximilians-Universität München, Theresienstrasse 41, 80333 Munich, Germany; 2 Istituto Nazionale di Geofisica e Vulcanologia, Sezione di Bologna, Viale Carlo Berti Pichat, 6/2, 40127 Bologna, Italy; 3 Istituto Nazionale di Geofisica e Vulcanologia, Sezione Roma 1, Via Di Vigna Murata 605, 00143 Rome, Italy; 4 School of Environment, Science Centre, University of Auckland, Building 302, 23 Symonds Street, Auckland Central, New Zealand; 5 Mercury NZ Ltd, 283 Vaughan Rd, Rotorua, 3010 New Zealand; 6 Dipartimento di Fisica e Geologia, Università degli Studi di Perugia, Perugia, Italy; 7 Lancaster Environment Centre, Lancaster University, Library Ave, Bailrigg, Lancaster, LA1 4YQ UK

**Keywords:** Geothermal field, Rotokawa, Degassing patterns, Hydrothermal alteration, Fluid flow, Thermal survey

## Abstract

Hydrothermal alteration exerts strong control on shallow permeability and degassing dynamics in geothermal systems. Here, we investigate how soil alteration influences near-surface gas flow by combining in situ petrophysical measurements with horizontal and vertical subsurface gas-concentration profiles at the Rotokawa geothermal field, New Zealand. These data are compared against unoccupied aerial system (UAS) thermal surveys of collapse structures across the field. Soil permeability at Rotokawa ranges from 8.7 × 10^–14^ to > 6.5 × 10^–13^ m^2^, highlighting strong meter- to decimeter-scale heterogeneity in shallow soil properties. Pumice-rich horizons are the main conduits for CO_2_ and CH_4_ flow, whereas clay-rich horizons locally act as semiconfining layers that promote CO_2_ accumulation and lateral diversion (leading to concentrations of > 4 × 10^4^ ppm). Since CO_2_ does not condense under near-surface conditions, the clay layers promote horizontal flow along permeable beds until gas encounters a high-permeability escape route or collapse-related discontinuity. Collapse structures locally disrupt and reorganize flow by acting as conduits or sinks that focus, capture, or redistribute gases near their margins. The gas profiles reveal patterns consistent with a shallow gas–steam decoupling zone in which steam condensation may contribute to sealing processes, as previously observed in steam-heated geothermal systems worldwide. These coupled effects of soil type and structural and alteration controls explain the spatial heterogeneity of surficial degassing at Rotokawa and provide a framework for interpreting evolving degassing patterns in similar steam-heated geothermal systems. In the context of the reported decrease in diffuse CO_2_ emissions at Rotokawa, progressive shallow sealing and gas refocusing may have contributed to apparent changes in emission patterns, alongside reservoir processes, recharge variability, environmental effects, and methodological uncertainty.

**Supplementary Information:**

The online version contains supplementary material available at 10.1007/s00445-026-02002-7.

## Introduction

The shallowest portion of active geothermal systems is characterized by a range of surface and soil conditions, with variations in temperature, soil strength, and the expression of hydrothermal features and alteration types. In this study, the term “soil” is used in a broad sense to refer to the upper unconsolidated volcanic surface materials, both in situ and anthropogenically reworked, that act as the medium for shallow fluid flow. The interplay among these characteristics results in complex shallow fluid flow patterns that are difficult to characterize. In addition, the surface of geothermal fields changes rapidly over time, with hydrothermal alteration profoundly modifying the physical and chemical properties of soils and rocks, affecting porosity, permeability, mechanical strength, mineral assemblages, and water content (Frolova et al. 2016; Heap et al. 2017; Mayer et al. 2017). Importantly, the dynamic interactions between rising geothermal fluids and near-surface environmental conditions include temperature and phase changes, steam condensation, variations in water content, mineral precipitation, and alteration reactions, which together exert a first-order control on gas and steam flow through the shallow subsurface.

Hydrothermal alteration of shallow volcanic soils and unconsolidated materials can influence subsurface fluid flow by locally increasing or decreasing porosity and permeability. The alteration products formed in low-temperature environments are strongly influenced by the composition of the parent material and its permeability and porosity (Browne 1978; Heap et al. 2017; Frolova et al. 2021). Porosity and permeability enhancement may occur via chemical leaching, dissolution and thermal fracturing, leading to more permeable, preferential flow pathways (e.g.,Browne 1978; Schöpa et al. 2011; Heap et al. 2017; Mayer et al. 2017). By contrast, precipitation of silica, sulfates, and clays during cooling, degassing, condensation, or oxidative near-surface reactions can reduce permeability and promote sealing (Renaut and Jones 2011; Sillitoe 2015; Pirajno 2020). These low-permeability zones may favor gas ponding and lateral diversion until gases encounter permeable discontinuities or discrete conduits. Where alteration instead promotes leaching, dissolution, or argillization, soils may lose cohesion and strength, favoring subsidence and collapse (Pola et al. 2014; Mayer et al. 2017; Rott et al. 2019; Frolova et al. 2021; Montanaro et al. 2023). Collapse pits are negative-relief landforms that develop in zones of focused hydrothermal fluid ascent, where sustained fluid flow promotes pervasive alteration and progressive mechanical weakening of the host rocks (Pola et al. 2014; Mayer et al. 2017; Frolova et al. 2021). This weakening can facilitate subsurface void formation and gravitational failure, ultimately leading to surface collapse. Together, sealing, weakening, and collapse processes reorganize the shallow soil, modifying preexisting depositional or pedological stratigraphy and creating permeability contrasts over a meter-scale across a field (Isaia et al. 2015; Frolova et al. 2016, 2019; Montanaro et al. 2023). This commonly leads to spatially heterogeneous pathways for gas and steam flow, as well as to changes in surface morphologies and patterns of diffuse and focused degassing (Ricci et al. 2015; Madonia et al. 2016; Montanaro et al. 2017, 2023; Semenkov et al. 2021).

Volcanic and geothermal terrains are important sites of CO_2_ soil degassing (e.g.,Chiodini et al. 1998; Viveiros et al. 2010; Bloomberg et al. 2014; Cardellini et al. 2017; Hughes et al. 2019; Bini et al. 2024; Klein et al. 2024; Yang et al. 2024). In these settings, it has been recognized that hydrothermal alteration can modulate degassing patterns at the meter scale and over temporal scales ranging from seasonal variability to long-term evolution, often associated with volcanic activity (e.g.,Schöpa et al. 2011; Tassi et al. 2013; Camarda et al. 2017; Heap et al. 2017; Mayer et al. 2017; Montanaro et al. 2017). However, how gases are redistributed within layered soils of contrasting permeability remains poorly constrained in the field, despite insights from laboratory experiments (Evans et al. 2001; Camarda et al. 2009). Field-based observations are often limited by the inherent complexity of natural stratigraphy, heterogeneous alteration, and anthropogenic modifications of the shallow subsurface.

Here, we present a field-based study at the Rotokawa geothermal field in Aotearoa (New Zealand). This field lies above an active high-temperature, gas-rich geothermal system and underwent extensive mining and industrial activities between ~ 1960 and 1990 (Jury 1984; Sinclair and Kear 1989; Giggenbach 1995; Bardsley and Williams 2017; Montanaro et al. 2023). Rotokawa also exhibits a strong spatial coupling among shallow permeability, soil alteration, and CO_2_ flux distribution in the Taupō Volcanic Zone, with marked spatial heterogeneity and significant temporal evolution in total CO_2_ emissions (Bloomberg et al. 2014; Yang et al. 2024). It therefore provides an excellent setting to investigate how soil type, alteration intensity, and structural features modulate fluid flow in the upper few meters of a geothermal system.

We examine geothermal fluid flow within the shallow subsurface at Rotokawa, focusing on how soil stratigraphy, alteration state, and local structures influence the accumulation of CO_2_, CH_4_, and H_2_O vapor. Previous studies at Rotokawa quantified field-scale CO_₂_ emissions, mapped soil and alteration types, and documented temporal changes in surface degassing (Bloomberg et al. 2014; Montanaro et al. 2023; Yang et al. 2024). However, the shallow pathways through which gases are redistributed within individual soil layers, along lithological contacts, and around collapse structures remain poorly resolved. We address this gap by integrating in situ petrophysical measurements, horizontal and vertical gas-concentration profiles, grain-size analyses, and high-resolution UAS visible and thermal surveys. This combined approach links centimeter- to decimeter-scale soil properties with meter-scale thermal and morphological expressions, allowing us to assess how stratigraphy, alteration, anthropogenic reworking, and collapse structures organize shallow gas and steam flow.

## Geological and hydrothermal settings

### Overview of the Rotokawa Geothermal Field

**Fig. 1:**
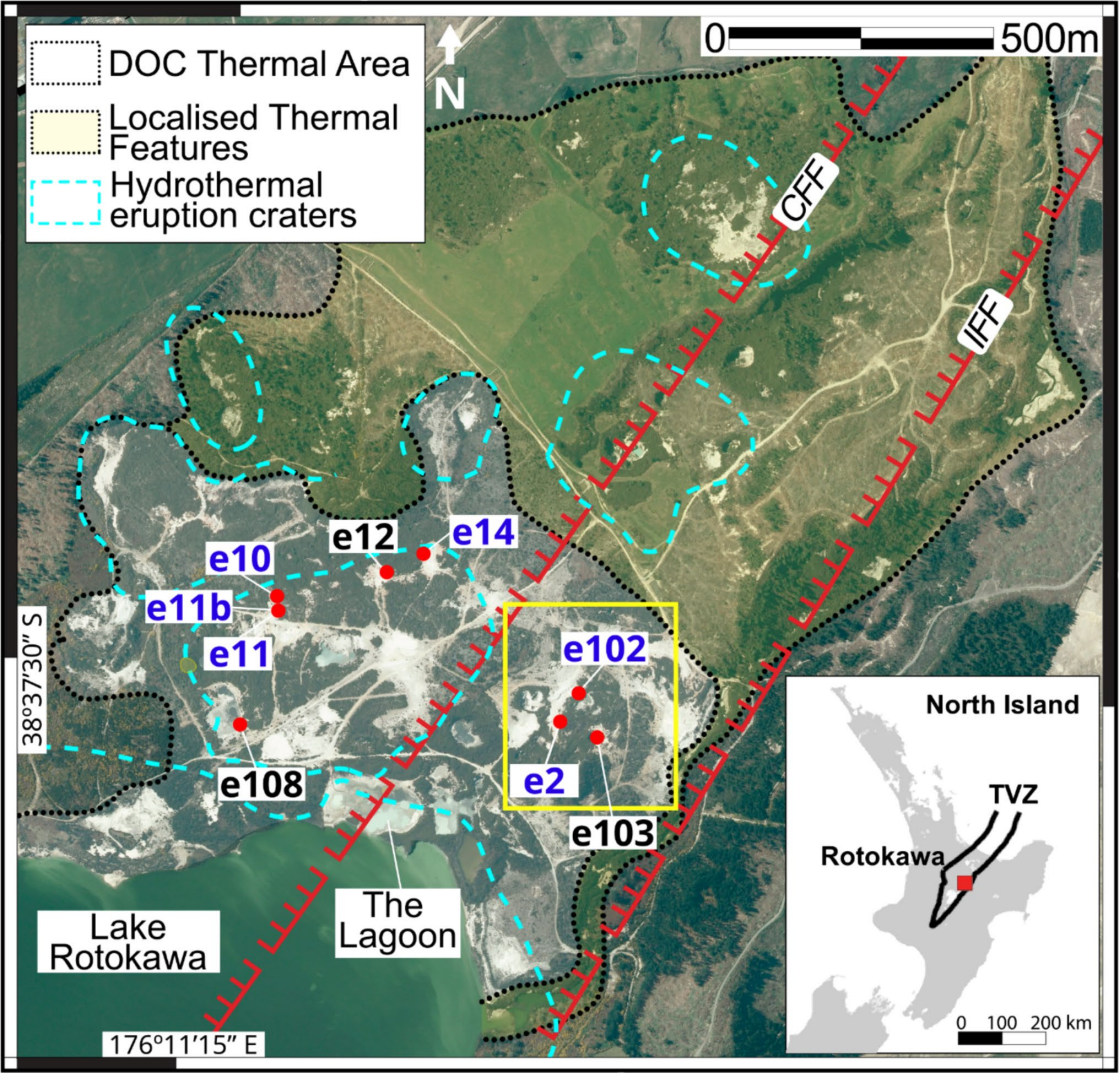
Aerial imagery of the Rotokawa thermal area, managed by the Department of Conservation (DOC-thermal area; modified from Montanaro et al. 2023), with the inset showing the central North Island of New Zealand and the location of the Rotokawa geothermal field (red square). Dotted lines outline the DOC-thermal area (gray shading) and the localized thermal features (yellow shading), which include steaming ground, mud pools, springs, fumaroles, encrusted grounds, and collapse structures. The cyan lines delimit the hydrothermal eruption craters (Bloomberg et al. 2014). The yellow square indicates the area analyzed with an unoccupied aerial system (UAS) survey. Major faults (CFF = central field fault; IFF = injection field fault) are shown in red. Sampling sites e2, e102, e103, e14, e12, e10, e11 and e11b, and e108 are shown, all situated within the ash- and pumice-rich surficial deposits of the Taupō Pumice Formation (in blue the sites where we performed in situ gas and petrophysical properties measurements and in black the ones where we performed only petrophysical properties measurements). Close up images of the different sampling sites are shown in Supplementary Fig. 1

The Rotokawa geothermal field is located in the North Island of New Zealand within the Taupō Volcanic Zone (TVZ; Fig. 1; Rowland and Simmons 2012) The field hosts a gas-rich high-temperature geothermal system (Giggenbach 1995) within a ~28 km^2^ low-resistivity anomaly (Risk 2000) with reservoir temperatures exceeding 350–360 °C at depths of ~2500 m (Hedenquist et al. 1988; Winick et al. 2011).

Structurally, the Rotokawa geothermal field is dominated by NNE-SSW to NE-SW-trending normal faults and extensional fractures, including the central field fault and several subsidiary structures (Rowland and Simmons 2012; McNamara et al. 2016). These structures act as conduits for deep fluid ascent and influence deep reservoir geometry and surface hydrothermal activity (Rowland and Sibson 2004; Hopp et al. 2020; Montanaro et al. 2023). Vertically, the system comprises three major aquifers separated by low-permeability units that reflect the combined effects of stratigraphy, alteration, and mineral precipitation (Winick et al. 2011; Sewell et al. 2012; McNamara et al. 2016). The deep aquifer (> 300 °C at ~ 1000 m depth) is a convecting, chloride-rich reservoir and forms the main geothermal production zone (Winick et al. 2011; Sewell et al. 2012; Addison et al. 2015; McNamara et al. 2016). Above it, the intermediate aquifer (300–1000 m) contains mixed cold meteoric water and rising geothermal fluids, resulting in highly acidic, corrosive conditions. The shallowest aquifer is dominated by meteoric groundwater with locally boiling zones and steam-bearing pockets, which directly feed surface manifestations and play a crucial role in shaping the near-surface alteration and degassing patterns (Glover and Mroczek 1995; Winick et al. 2011; Addison et al. 2015; Sewell et al. 2015; McNamara et al. 2016).Interaction between shallow groundwater and CO_2_ and H_2_S rich boiling gases produces acid–sulfate fluids in the main up flow zones, while bicarbonate fluids accumulate along the outflow margins. These acidic fluids promote dissolution of the in-situ Taupo Pumice, progressively reducing grain size, cohesion and mechanical strength. Cooling, condensation and changes in fluid pH also promote precipitation of secondary minerals including native sulfur, kaolinite, smectite, opal, and minor alunite (Krupp and Seward 1987; Brooks-Clarke 2021; Chambefort 2021; Simpson et al. 2021; Montanaro et al. 2023).

Surficial thermal features are concentrated within the Department of Conservation (DOC) thermal area (Fig. 1), where pumice-rich deposits of the Taupō Pumice Formation host the acidic Lake Rotokawa (pH ~ 2), numerous collapse pits, fumarolic fields, steaming ground, altered hard-ground surfaces, sulfur crusts and stromatolite-bearing terraces (Collar 1985; Krupp and Seward 1987; Montanaro et al. 2023).

The central part of the Rotokawa thermal area was significantly modified by intensive sulfur mining between the 1960 s and the early 1990 s, which altered the shallow hydrology and permeability, reshaped collapse features, and locally removed alteration caps (Jury 1984; Sinclair and Kear 1989; Bloomberg et al. 2014; Bardsley and Williams 2017; Montanaro et al. 2023). CO_2_ flux surveys conducted at the Rotokawa geothermal field provide key constraints on the magnitude and evolution of natural degassing. Bloomberg et al. (2014) estimated CO_2_ emissions of 441 t d^−1^ and H_2_S emissions of 31 Mg d^−1^. Yang et al. (2024) reported a total CO_2_ emission of ~ 345 t d^−1^ in 2023, based on repeated surveys at 508 identical locations. Although these values suggest a decrease, the overlapping emission ranges and sensitivity of diffuse fluxes to environmental and methodological factors mean that interannual to decadal trends should be interpreted cautiously (Yang et al. 2024).

### Surficial soil types

A range of unconsolidated surficial deposits occur at Rotokawa including undisturbed primary pumice pyroclastic deposits interlayered with reworked, altered, or anthropogenically modified soils previously mapped and described in detail by Montanaro et al. (2023). These are affected in diverse ways by hydrothermal alteration, boiling, collapse processes, and historical mining activities. Here, we use this established classification as geological context for the new petrophysical, grain size, gas profile, and UAS datasets presented in this study, distinguishing between primary Taupō Pumice units and reworked deposits (Supplementary Table 1). Undisturbed soils at Rotokawa are formed in the Taupō Pumice Formation (T). These include pumice-rich fall deposits (T1–T3), ranging from relatively unaltered, silt- to sand-rich layers with granule- to boulder-sized pumice clasts (T1–T2) to more intensely altered, clay-enriched horizons with heavily altered pumice clasts (T3). Other primary units are pyroclastic flow deposits (T4 and T5), which are coarse-grained, pumice-rich layers containing abundant lapilli-sized clasts and locally intense sulfur or silica alteration. They are commonly pebble- to cobble-rich and may contain centimeter-sized sulfur nodules and crystals; T5 is generally more altered and with slightly finer grains than T4. These coarse units typically exhibit among the highest measured permeabilities, although T5 is strongly hydrothermally altered and may develop partial sealing, making it, together with T3, one of the two most altered soil types within the undisturbed sequence.

Reworked soils encompass several subtypes that reflect mechanical excavation, natural or anthropogenically induced collapse, fluid focusing, or surface sediment reworking via alluvial processes. Excavated materials (E1–E3) derive from extensive sulfur mining activities and consist of heterogeneous mixtures of pumice, altered tephra, and locally clay-rich fragments, typically showing sharp contacts with the undisturbed layers. Clay-rich reworked units (C1 and C2) form distinct, laterally continuous horizons that separate the primary pumice deposits from the excavated materials. These layers are clay-enriched and commonly water-saturated; where sufficiently continuous, they can locally reduce permeability and act as semiconfining horizons. M-type layers are clay-rich, plastic soils forming in excavated depressions influenced by boiling mud pools and persistent steam condensation. These show very low permeability, high water content, and intense acid–sulfate alteration. O-type soils consist of alternating silt and sandy beds containing root fragments, organic matter, and clear signs of oxidation. They typically occur away from active geothermal features and retain higher permeability less reworked textures. Brecciated units (Br) comprise coarse, angular debris produced by collapse, subsidence, or repeated hydrothermal disruption and exhibit highly variable grain size and permeability depending on alteration and compaction.

## Methodology

To understand the relationship between fluid flow patterns and soil alteration, we combined in situ petrophysical measurements, shallow gas concentration profiles, and grain size analyses for the main soil types identified by Montanaro et al. (2023). These field-based measurements were integrated with unoccupied aerial system (UAS) visible and thermal surveys of selected collapse structures, allowing the layer-scale controls on permeability, mechanical weakening, and gas migration to be evaluated in relation to pit-scale morphology and thermal activity. This multimethod approach allows us to link permeability, mechanical weakening, and textural variability to spatial variations in the migration of CO_2_, CH_4_, and H_2_O.

### Soil petrophysical and mechanical measurements

During a field campaign in February 2022, Montanaro et al. (2023) measured permeability, humidity, compressive and shear strength, as well as soil temperature at sites representative of all surficial soil layers at Rotokawa. To ensure temporal comparability, we resampled and remeasured the same locations investigated by Montanaro et al. (2023), which additionally enables us to assess the short-term evolution of the soil properties. A total of 36 quasi-undisturbed soil-cores were collected using stainless steel cylinders (diameter = 7.2 cm; length/height = 6.1 cm; Supplementary Fig. 2). Cylinders were inserted vertically and/or horizontally into the soil layer of interest, depending on the exposure, and excess material was carefully trimmed from both ends. Complete filling of the cylinder was verified visually to avoid marginal voids, which could promote preferential air leakage and artificially high permeability values. After extraction, samples were immediately sealed with plastic wrap to preserve their natural water content and pore structure and were analyzed within a few hours to minimize any desaturation and oxidation effects. Permeability and moisture content measurements were therefore performed on centimeter-scale, quasi-undisturbed cores shortly after sampling. These data are used primarily to compare relative petrophysical contrasts among adjacent soil layers, whereas mechanical strength, gas concentration, and temperature measurements were performed directly on the outcrop walls.

Air permeability was measured using a PL-300 soil permeameter manufactured by Umwelt-Geräte-Technik GmbH (UGT; Umwelt-Geräte-Technik 2014, 2019) that applies Darcy’s law to determine volumetric gas flow through the connected pores network. The instrument has a wide effective measurement range of air permeability, from 5.6 × 10^–16^ to 6.5 × 10^–13^ m^2^ (Montanaro et al. 2017, 2023). Values exceeding the upper measurement limit were treated as minimum estimates and reported as > 6.5 × 10^–13^ m^2^. The permeability calculation is performed internally by the instrument following the manufacturer’s formulation. The instrument was calibrated in the laboratory prior to the field campaign using the standards provided by the manufacturer. The reported values therefore represent effective air permeability under the field-humidity conditions at the time of sampling, rather than dry intrinsic permeability. Soil humidity was measured using a time–domain reflectometry (TDR) sensor integrated with the soil permeameter system (Umwelt-Geräte-Technik 2014, 2019). The TDR probe was inserted adjacent to the sampling cylinder to ensure consistency between hydraulic and petrophysical measurements.

**Fig. 2:**
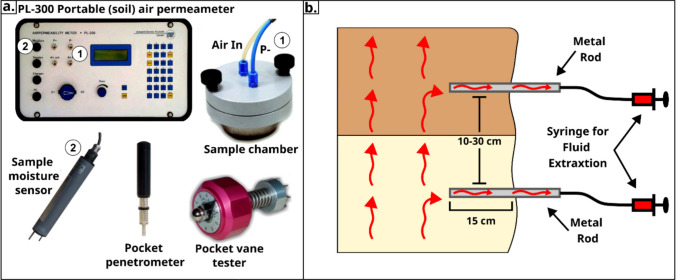
Field methods used to characterize shallow soil properties and subsurface gas flow. **a** In situ petrophysical measurements, including air permeability via PL-300 with integrated moisture and temperature sensors, and pocket penetrometer and shear vane tests for compressive and shear strength. Numbers 1 and 2 indicate, respectively, where the sample chamber and the sample moisture sensor are connected. **b** Schematic illustration of horizontal and vertical subsurface gas–concentrations profiling. A hollow metallic probe was inserted 15 cm into the soil at 15–30-cm intervals, and gas extracted with a syringe was analyzed for CO_2_, CH_4_, and H_2_O vapor concentration

Mechanical properties were determined via compressive strength (pocket penetrometer manufactured by Royal Eijkelkamp; 0–5 kg/cm^2^; resolution 0.1 kg/cm^2^) and shear strength (hand shear vane manufactured by Gilson Company Inc.; 0–5 kg/cm^2^ measurement range; resolution 0.1 kg/cm^2^) both performed in situ on the outcropping wall. To minimize local heterogeneities, compressive strength was measured 4–5 times per site and shear strength 3–4 times, allowing us to compute representative mean values and standard deviations (Farquhar 2001; Mir 2021; Mousavi et al. 2021). Soil temperature at 15-cm depth was measured using a K-type thermocouple inserted directly at the sampling location (Fig. 2a). The depth of 15 cm was chosen for consistency with previous soil-temperature, CO_2_ flux, and soil characterization studies at Rotokawa geothermal field (Bloomberg et al. 2014; Montanaro et al. 2023; Yang et al. 2024).

### Gas concentration measurements

In areas where high diffuse CO_2_ degassing was documented in previous surveys (Bloomberg et al. 2014; Yang et al. 2024), we investigated subsurface gas flow by measuring the in situ interstitial concentrations of CO_2_, CH_4_, and H_2_O vapor along shallow horizontal and vertical profiles. We used shallow gas probing techniques with fixed-depth lateral or vertical insertion as applied to volcanic and hydrothermal environments (Camarda et al. 2006, 2009). After scraping the surficial soil patina or opening a shallow trench, a metallic hollow rod connected to a syringe was inserted laterally 15 cm into the exposed soil wall at each sampling point. Sampling points were arranged along horizontal or vertical profiles at 15–30-cm intervals, depending on soil conditions and accessibility (Fig. 2b). This depth corresponds to the zone where gas–steam decoupling, condensation, and lateral migration are most commonly observed in hydrothermally altered soils (Camarda et al. 2006). The creation of the soil trench inevitably introduces a local perturbation to gas diffusion, potentially promoting lateral gas escape near the surface. Sampling at 15 cm was therefore chosen to reduce the influence of artificial near-surface pathways generated during trench opening, while remaining consistent with previous soil-temperature, CO_₂_ flux, and soil characterization studies at Rotokawa. Gas was extracted using a syringe and immediately injected into a portable infrared gas analyzer to quantify CO_2_, CH_4_, and H_2_O vapor concentrations. Carbon dioxide (CO_2_) and water vapor (H_2_O) concentrations were determined by an integrated LICOR Li-840A infrared spectrophotometer (0–20,000 ppm CO_2_ measurement range; 0–60 mmol/mol H_2_O, 1.5% accuracy). The instrument’s internal software extrapolates concentrations exceeding the 0–20,000 ppm calibration range based on the coefficients established within this range. Methane (CH_4_) concentration was measured using a tunable diode laser coupled with a Herriot multipass cell (0–20,000 ppm, 1% accuracy). Gas was continuously drawn through the system by a membrane pump connected via silicone tubing at a flow rate of ~ 4 L/min.

Profiles ranged from 60 cm to more than 2 m in length, depending on the exposure and the continuity of the investigated soil layer. The combined horizontal and vertical profiles were designed to resolve gas accumulation and depletion across individual soil layers, lithological contacts, and collapse-related structures at centimeter- to decimeter-scale resolution. This approach is conceptually similar to laboratory-based gas transport experiments that investigate gas redistribution across layered porous media (e.g., Camarda et al. 2009). Temperature was recorded at each single sampling point using a miniature thermocouple. Similar multipoint shallow gas profiles have been used to detect permeability contrasts and hydrothermal fluid pathways at volcanic systems such as Solfatara (Italy), Vulcano (Italy), and La Soufrière de Guadeloupe (Ricci et al. 2015; De Landro et al. 2017; Gaudin et al. 2017). These measurements therefore provide a field-based method to compare fine-scale gas accumulation patterns with local stratigraphy, distribution, alteration state, and petrophysical contrast.

### Grain size distribution analyses

To characterize the textural controls on permeability and fluid flow, we analyzed the grain size distribution of selected samples representing key soil types from the primary Taupō Pumice units and clay-rich reworked horizons. Samples were dried overnight at 60 °C to determine their initial dry weight. Wet sieving was then performed using mesh sizes of 1 mm, 710 μm, 500 μm, and 355 μm; particles finer than 355 μm were collected in glass bulbs and allowed to settle for at least 24 h. Each fraction was then oven-dried and weighed to determine the mass distribution of grains > 355 μm. For the finer fraction (< 355 μm), grain size distribution was determined by laser diffraction using a Bettersizer S3 Plus instrument, following standard operating procedures (Bettersizer 2022). The fine particles were dispersed in distilled water, with a magnetic stirrer and sodium phosphate used to prevent aggregation and clumping. Fractions coarser than 1 mm were additionally separated via dry sieving using mesh sizes from 16 to 355 μm to capture coarser pumice and breccia clasts common in T4–T5 soils. The resulting grain size distributions were used to compare textural contrasts among the sampled layers and to support interpretation of permeability and gas accumulation patterns.

### UAS surveys

We conducted visible and thermal infrared UAS surveys to characterize the main thermal area of the Rotokawa geothermal field, including selected collapse structures. The UAS surveys were designed to bridge the scale gap between point-scale field measurements and the field-scale expression of hydrothermal activity. Petrophysical measurements and shallow gas profiles quantify the permeability, strength, and gas flow behavior of individual soil layers, whereas the UAS-derived visible and thermal models reveal how these layer-scale controls are expressed at the scale of collapse structures. We therefore use the UAS data as a spatial framework for interpreting the geomorphic and thermal expression of shallow permeability contrasts, alteration intensity, and focused fluid flow. For the main collapse structures, we flew at approximately 15 m above ground level using a DJI Mavic 3 T. At this flight height, the spatial resolution was approximately 1 cm/pixel for the visible imagery and approximately 2 cm/pixel for the thermal imagery. The overview of the broader area was obtained at a flight height of approximately 120 m above ground level, resulting in a thermal spatial resolution of approximately 16 cm/pixel. Thus, the maximum temperatures obtained may be attenuated by surrounding colder regions that fall in the same pixel. The 3-D geometry reconstruction was performed using the Agisoft Metashape Professional® software package (version 2.2.2), based on the SfM–MVS algorithm, and the CloudCompare open-source software (www.cloudcompare.org, version 2.13.2). The photogrammetric analyses followed the same SfM-MVS workflow adopted by Civico et al. (2022, 2024). For the analysis of single thermal frames, we used the DJI Thermal Analysis Tool 3 (version 3.4.0).

## Results

**Fig. 3:**
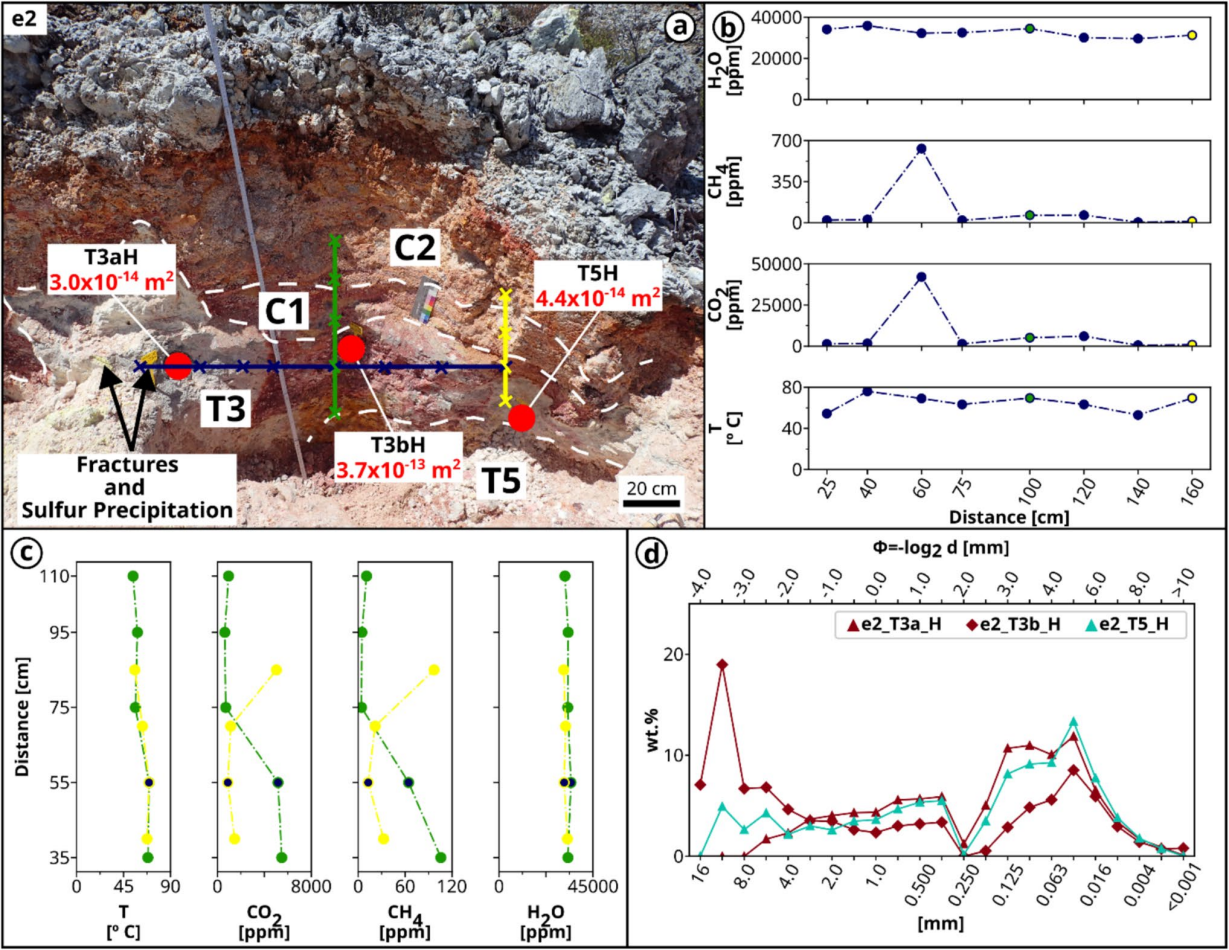
Stratigraphy, petrophysical measurements, and subsurface gas-accumulation profiles at site e2. **a** Overview showing the positions of horizontal and vertical gas sampling transects and locations of petrophysical samples within the T3–C1–C2 sequence and adjacent T5. Fractures and sulfur precipitation are visible at the surface. **b** CO_2_, CH_4_ and H_2_O vapor concentrations and temperature along the horizontal transect, with overlapping points from the vertical profiles (green and yellow data points; the colors correspond to the profiles in panel **a**). **c** Vertical gas-accumulation profiles showing strong CO_2_ and CH_4_ enrichment at the T3–C1 contact and suppression within clay-rich horizons, with overlapping points for the horizontal tansect (blue points). The yellow and green data points correspond to the colored profiles in panel **a**. **d** Grain size distribution of selected T3 and T5 samples illustrating fine-dominated altered T3 and coarser fractions in T5 (colour indicates the type of soil)

**Fig. 4:**
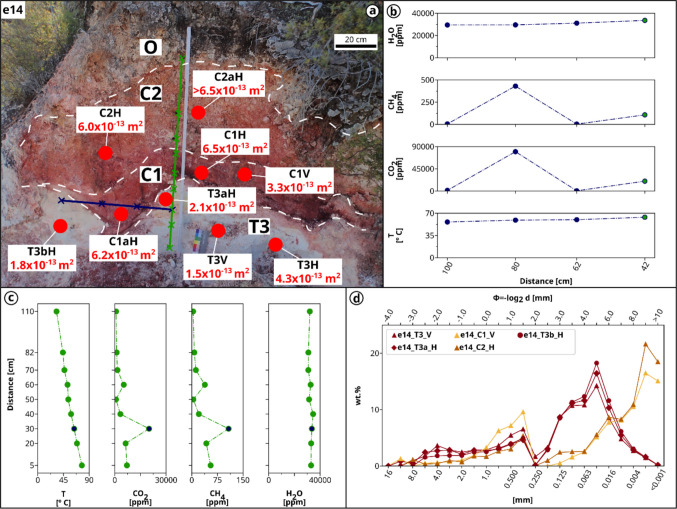
Stratigraphy, petrophysical measurements, and subsurface gas-accumulation profiles at site e14. **a** Overview image of the T3–C1–C2–O sequence on the northern margin of a hydrothermal-eruption crater, showing locations of petrophysical samples (red dots with permeabilities; H and V indicate sampling orientation) and horizontal and vertical gas-sampling transects (blue and green lines). **b** CO_2_, CH_4_, and H_2_O vapor and temperature along the horizontal transect highlighting strong CO_₂_ enrichment at the T3–C1 contact (green data point indicates the overlapping point between the two profiles; the colors correspond to the profiles in panel **a**). **c** Vertical gas-accumulation profile showing CO_2_ suppression within the clay-rich C1–C2 interval (dark blue data point indicates the overlapping point between the two profiles; the colors correspond to the profiles in panel **a**). **d** Grain size distributions of selected samples illustrating fine T3 fractions and clay-dominated C1–C2 horizons (color indicates the type of soil)

**Fig. 5:**
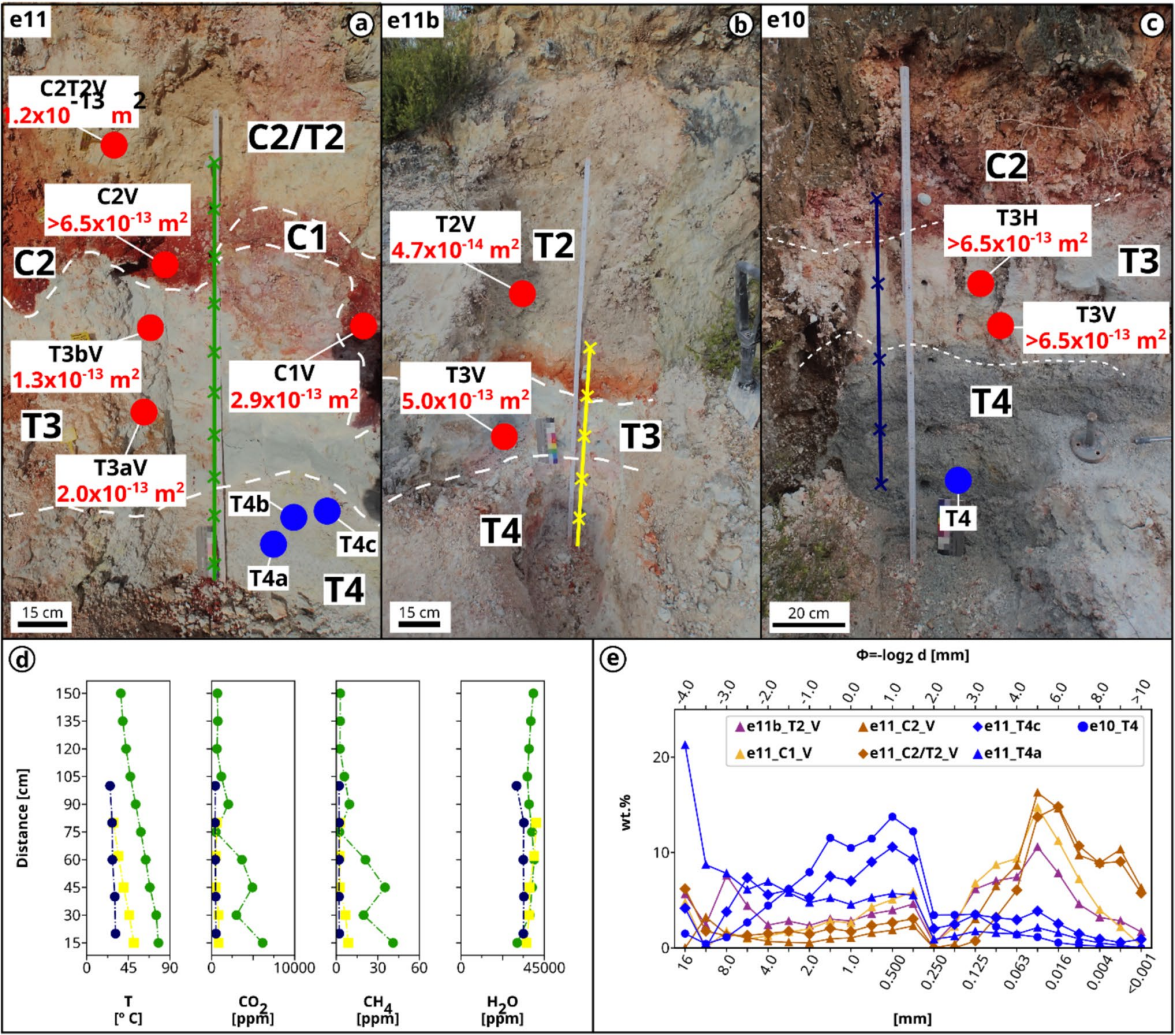
Stratigraphy, petrophysical measurements, and subsurface gas-accumulation profiles at sites e11, e11b, and e10. **a** Overview image of the T4–T3–C1–C2–C2/T2 sequence at the highly altered e11 transect, locations of petrophysical samples (red dots with permeabilities; H and V indicate sampling orientation) and vertical gas-sampling transect (green line). **b** Overview image of the T4–T3–T2 sequence at the low-alteration e11b site, locations of petrophysical samples (red dots with permeabilities; H and V indicate sampling orientation) and vertical gas-sampling transect (yellow line). **c** Overview image of the T4–T3–C2 sequence at site e10, locations of petrophysical samples (red dots with permeabilities; H and V indicate sampling orientation) and vertical gas-sampling transect (blue line). **d** Vertical gas-accumulation profiles showing high values of CO_2_ and CH_4_ accumulation at the T4 and T3 layers of site e11. Gas accumulation decreases rapidly at the boundary between T3 and C1/C2 (the colors of the transects correspond to panels **a**, **b,** and **c**). **e** Grain size distributions of selected samples illustrating coarse T4 fractions, finer T2 fractions and clay-dominated C1–C2 horizons (color indicates the type of soil)

**Fig. 6:**
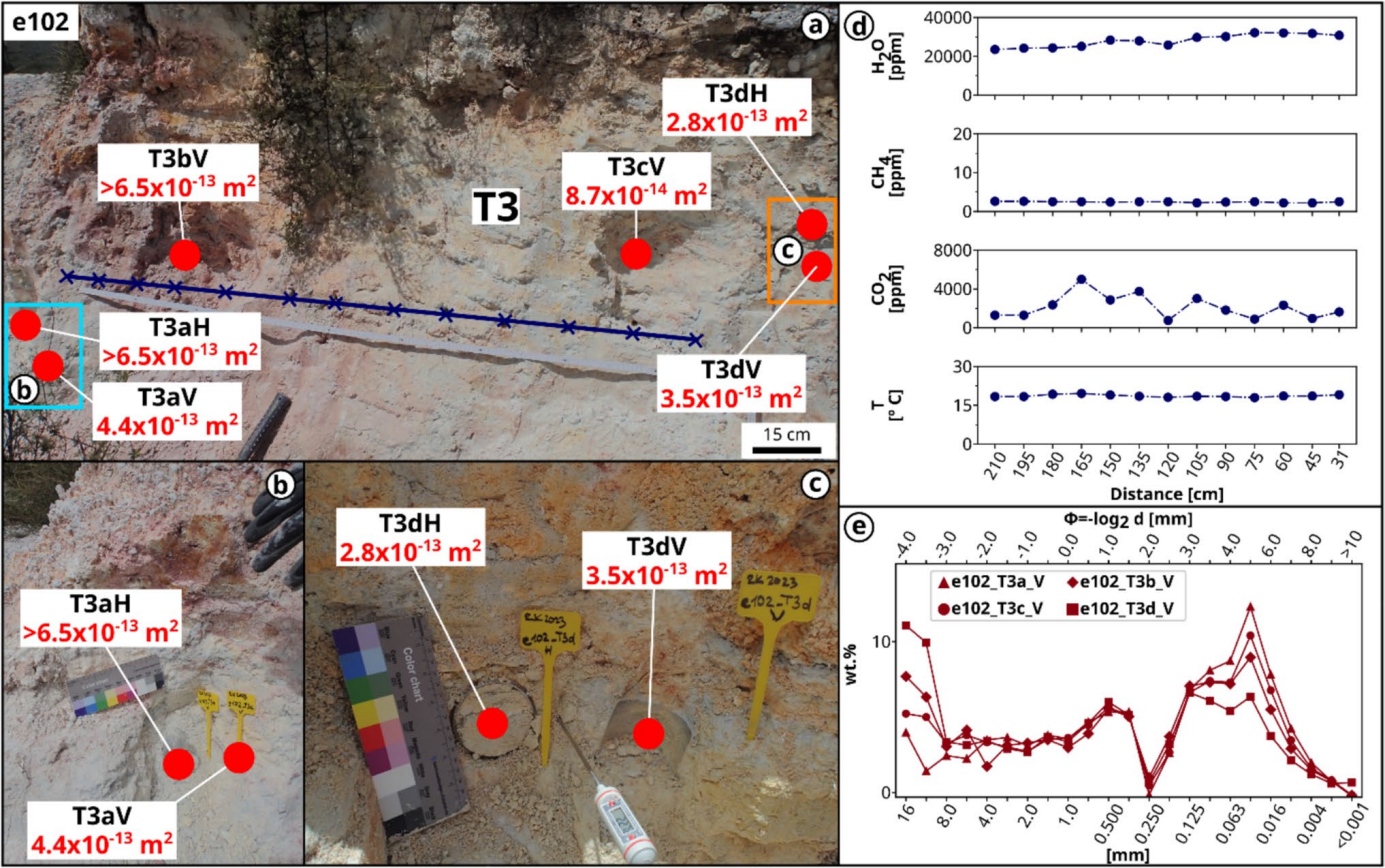
Stratigraphy, petrophysical measurements and subsurface gas-accumulation profile at sites e102. **a** Overview image of site T3 layer showing various degrees of alteration, locations of petrophysical samples (red dots with permeabilities; H and V indicate sampling orientation) and horizontal gas-sampling transect (blue line). **b** Close-up image of the sampling location of samples T3aH and T3aV. Scale card measures 18 cm in length. **c** Close-up image of sampling location of samples T3cH and T3dV. **d** CO_2_, CH_4_, and H_2_O vapor and temperature along the horizontal transect highlighting CO_₂_ enrichment in the highly altered portion of T3, with a rapid decrease towards the collapse structure located left of the transect. **e** Grain size distributions of selected samples illustrating fine-dominated T3 samples, with the amount of fines increasing with alteration (color indicates the type of soil)

We analyzed the petrophysical properties of soil from nine sites across the main thermal area of the Rotokawa geothermal field (namely, e108, e11, e11b, e10, e12, e14, e2, e102, and e103 as shown in Fig. 1 and Supplementary Fig. 1). The full dataset is reported in Supplementary Table 2 and shown in Figs. 3, 4, 5, and 6. In total, we obtained 36 measurements of permeability and humidity and 35 measurements of compressive and shear strength. The sample orientation is indicated by “H” (horizontal) and “V” (vertical). We also measured CO_2_, CH_4_, and H_2_O vapor concentrations along three horizontal and six vertical profiles (sites e2, e14, e11, e11b, e10, and e102). Grain size analyses were performed on 19 soil samples from these profiles.

### Site e2

Site e2 hosts a well-defined alteration halo around a small fumarole (Figs. 1 and 3; Supplementary Fig. 1). The sequence consists of a coarse T5 layer overlain by an altered T3 layer and capped by clay-rich C1–C2 layers. Alteration intensity increases towards the fumarole, with a red/brown, stiffer T3 becoming white and friable near the halo center. Permeabilities span 3 × 10^–14^–3.7 × 10^–13^ m^2^, humidity of 15**–**34.8%, with elevated temperatures (59–73.3 °C), and moderate compressive and shear strength (2.5 to 3.9 kg/cm^2^ and 2.6 to 3 kg/cm^2^, respectively; Supplementary Table 2). Three samples were collected from T3 and T5 (T3aH, T3bH, and T5H). The altered T3 and T5 at this site are dominated by fines (125–16 μm), whereas the less altered T3 (T3bH) also contains coarser pumice clasts (~ 8 mm) (Fig. 3d).

Gas profiling (both vertical and horizontal) reveals high CO_2_ concentrations, ranging from 551.6 to 42,112.65 ppm, with CH_4_ values of 4.2–631.7 ppm, and H_2_O vapor of 29,578–35,872 ppm. Temperatures varied from 53 to 76 °C (Supplementary Table 2 and Fig. 3b and c). The highest gas concentrations occur at the T3–C1 interface and near the fumarole.

### Site e14

Site e14 lies on the northern edge of a large hydrothermal eruption crater (Fig. 1; Supplementary Fig. 1). The stratigraphy starts with a basal pale grey T3 layer, transitioning into a red, clay-rich C1 layer, overlain by a thick C2 unit and an organic-rich O horizon (Fig. 4a). T3 samples (T3V, T3H, T3aH, and T3bH) have permeabilities of 1.8 × 10^–13^–4.3 × 10^–13^ m^2^, humidity of 16.2–19.9%, temperatures of 59–63.1 °C, high compressive strength variability (from 1.4 to > 5 kg/cm^2^), and more stable shear strength values (from 2.2 to 2.4 kg/cm^2^). Clay-rich C1 and C2 samples (C1H, C1V, C1aH, C2H, and C2aH) show high permeability (3.3 × 10^–13^– > 6.5 × 10^–13^ m^2^), low-mid humidity (14–41.2%) and temperatures (36–62.2 °C), and lower strength (compressive strength from 0.7 to 1.1 kg/cm^2^ and shear strength from 1.8 to 2.4 kg/cm^2^; Supplementary Table 2 and Fig. 4a). Thus, despite their clay-rich texture, C1–C2 layers at this site do not show systematically lower measured air permeability than the underlying T3 samples. T3 samples (T3V, T3aH, and T3bH) are dominated by 125–16 μm particles with a mode at 32 μm, whereas C1–C2 layers (C1V and C2H) are composed almost entirely of < 4 μm clays, consistent with their intense alteration and plasticity.

Gas concentrations along horizontal and vertical profiles are highly variable, with CO_2_ ranging from 523 to 79,790 ppm, CH_4_ of 3–431 ppm, and H_2_O vapor of 29,476–34,575 ppm. Temperatures varied from 32.8 to 77.3 °C (Fig. 4b and c). The highest gas concentrations occur near sharp T3–C1 contacts.

### Sites e11, e11b, and e10

These three sites lie in the same crater as e14 and near a small collapse structure (Figs. 1, and 5). Site e11 and e11b are adjacent, with e11b representing the more highly altered profile, while site e10 lies approximately 20 m to the north (Fig. 1; Supplementary Fig. 1). All three sections expose coarse T4 deposits at their base (not cored), overlain by a pale gray to brownish T3 layer. In the upper part of e11, clay-rich layers C1 and C2 grade downward into weakly altered material atop T2/C2. A thick T2 layer dominates the upper e11b profile, whereas e10 shows a clay-rich C2 layer horizon near the top (Fig. 5a–c). T3 samples from all sites (T3aV, T3bV, T3V, T3H, and T3V) show permeabilities between 1.3 × 10^–13^ and > 6.5 × 10^–13^ m^2^, humidity of 16.5–27.4%, temperatures of 28.9–57.5 °C, and strengths ranging from moderate to high (compressive strength from 1.2 to > 5 kg/cm^2^ and shear strength from 2.0 to 2.6 kg/cm^2^; Supplementary Table 2 and Fig. 5a–c). The T2 layer at site e11b (T2V) displays a relatively low permeability (4.7 × 10^–14^ m^2^), low humidity (20.6%), and temperature (30.1 °C), and mid-strength values (compressive strength of 3.3 kg/cm^2^ and shear strength of 2.7 kg/cm^2^; Supplementary Table 2 and Fig. 5b). Clay-rich C1–C2 samples of site e11 (C1V, C2V, and C2T2V) exhibit permeabilities of 1.2 × 10^–13^– > 6.5 × 10^–13^ m^2^, humidity of 17–34.2%, temperatures of 33.5–44.1 °C, and low-mid strength (compressive strength from 0.5 to 1.2 kg/cm^2^ and shear strength from 1.9 to 2.6 kg/cm^2^; Supplementary Table 2 and Fig. 5a). Grain size analyses confirm fine-grained C1 and T2 layers (peaks at 32 μm), very fine C2–C2/T2 (< 16 μm), and coarse T4 samples, dominated by > 250 μm particles (Fig. 5e).

Gas measurements along three vertical profiles (one per site) show moderate CO_2_ concentrations (434–6142 ppm), low CH_4_ (2–41 ppm), and H_2_O vapor (30,053 to 40,707 ppm), and significant temperature variability (25–78 °C). Gas decreases sharply within clay-rich intervals (C1 and C2 horizons; Fig. 5a–d).

### Site e102

Site e102 is located near large collapse structures and exposes a laterally variable T3 layer with strong alteration gradients (Figs. 1, and 6a; Supplementary Fig. 1). Samples from the halo center and more proximal to the pit (T3aH and T3aV) are white, friable, and highly altered (Fig. 6a, and b). One sample (T3bV) was collected at the halo margin from a red-stained T3 horizon. Moving laterally into the off-white T3 unit, a second sample was taken (T3cV; Fig. 6a). Further from the alteration zone, two additional samples were obtained from moderately altered yellow-brown T3 (T3dH and T3dV; Fig. 6a, and c). Permeability ranges from 8.7 × 10^–14^ to > 6.5 × 10^–13^ m^2^, humidity from 12 to 20.2%, with low temperatures (20.1–22.9 °C), and low to high strength (compressive strength from 1.5 to 3.7 kg/cm^2^ and shear strength of 2.4 to 4.2 kg/cm^2^; Supplementary Table 2 and Fig. 6a). Strength increases away from the collapse feature. Grain size distribution indicates poor sorting, with coarse clasts (16–8 mm) more abundant in weakly altered samples (e.g., T3dV), while fine fractions (125–16 μm) dominate overall (Fig. 6e).

A horizontal gas profile shows CO_2_ concentrations of 758.5–5000 ppm, very low CH_4_ (2.2–2.6 ppm), and H_2_O vapor of 23,514–32,194 ppm. Temperatures vary from 18 to 19.6 °C (Fig. 6d). Gas concentrations increase toward highly altered T3 but drop sharply near the collapse margin.

### Sites e108, e12 and e103

Across these three sites, nine samples were collected (T3V, T3H, C1H, C2H, C2V, T2V, T3bV, T3aH, and T3bH). T3 samples show high permeability (2 × 10^–13^– > 6.5 × 10^–13^ m^2^), humidity of 8–48.6%, temperatures of 26.2–53.9 °C, and a wide strength range (compressive strength from 0.3 to >5 kg/cm^2^ and shear strength from 1.0 to 3.9 kg/cm^2^; Supplementary Table 2). Clay-rich C1–C2 samples display variable permeability (3.8 × 10^–13^– > 6.5 × 10^–13^ m^2^), low humidity (17–19.5%), and temperatures (23.6–24.9 °C), with low to moderate strength (compressive strength from 0.2 to 3.0 kg/cm^2^ and shear strength from 1.3 to 3.6 kg/cm^2^; Supplementary Table 2). The T2 sample at e103 exhibits a high permeability (3.7 × 10^–13^), low humidity (16.3%) and temperature (19.2°C), and moderate strength values (~3.4 kg/cm^2^; Supplementary Table 2). No gas profiling was performed at these sites, which are therefore used primarily to extend the petrophysical comparison among soil units.

### UAS thermal imaging

**Fig. 7:**
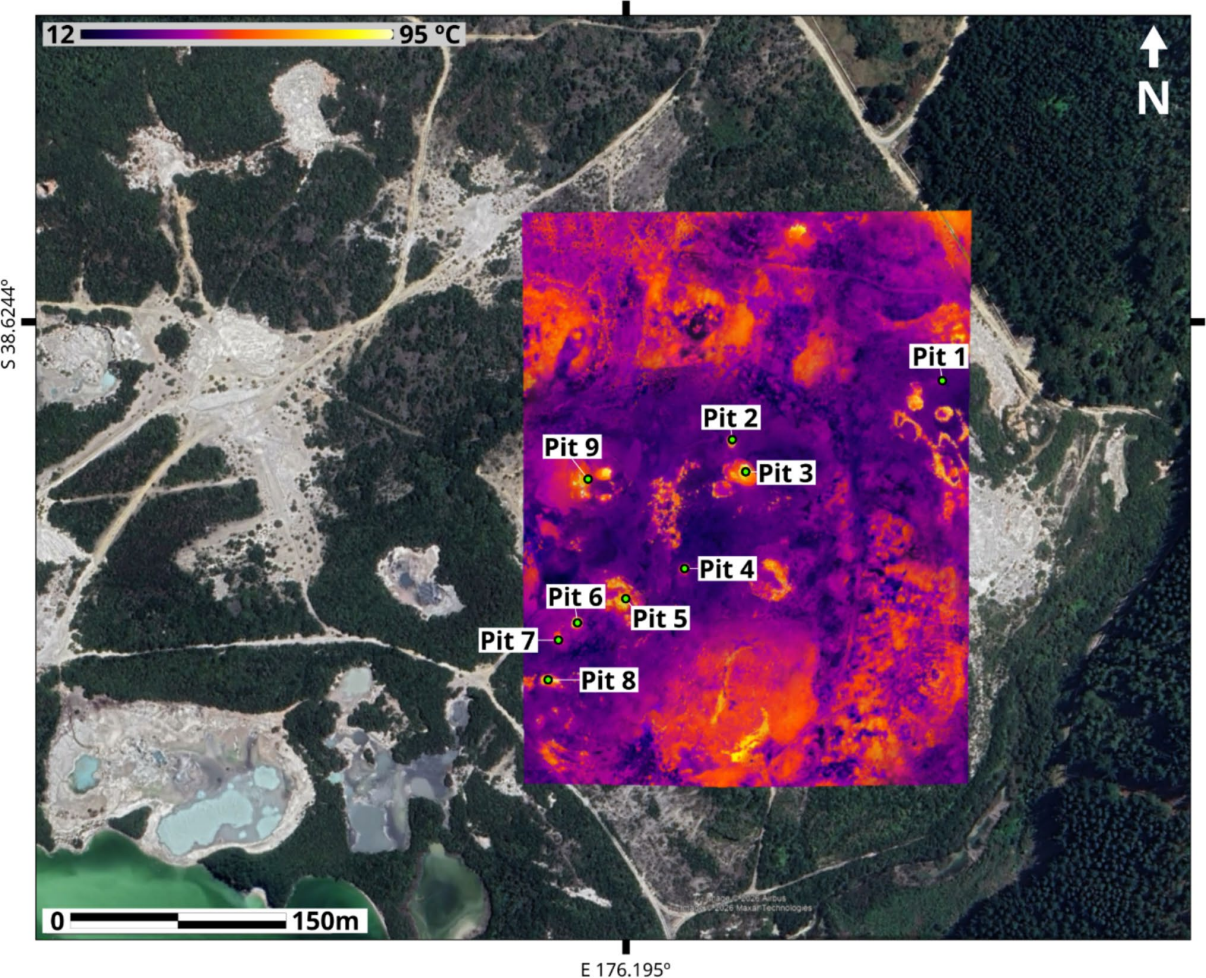
Google Earth image of the central part of the Rotokawa geothermal field and overlain by thermal infrared UAS survey image (yellow square in Fig. 1; colder areas in dark colors and warmer areas highlighted with brighter reds and yellows). The green dots indicate the location of the 9 analyzed pits

**Fig. 8:**
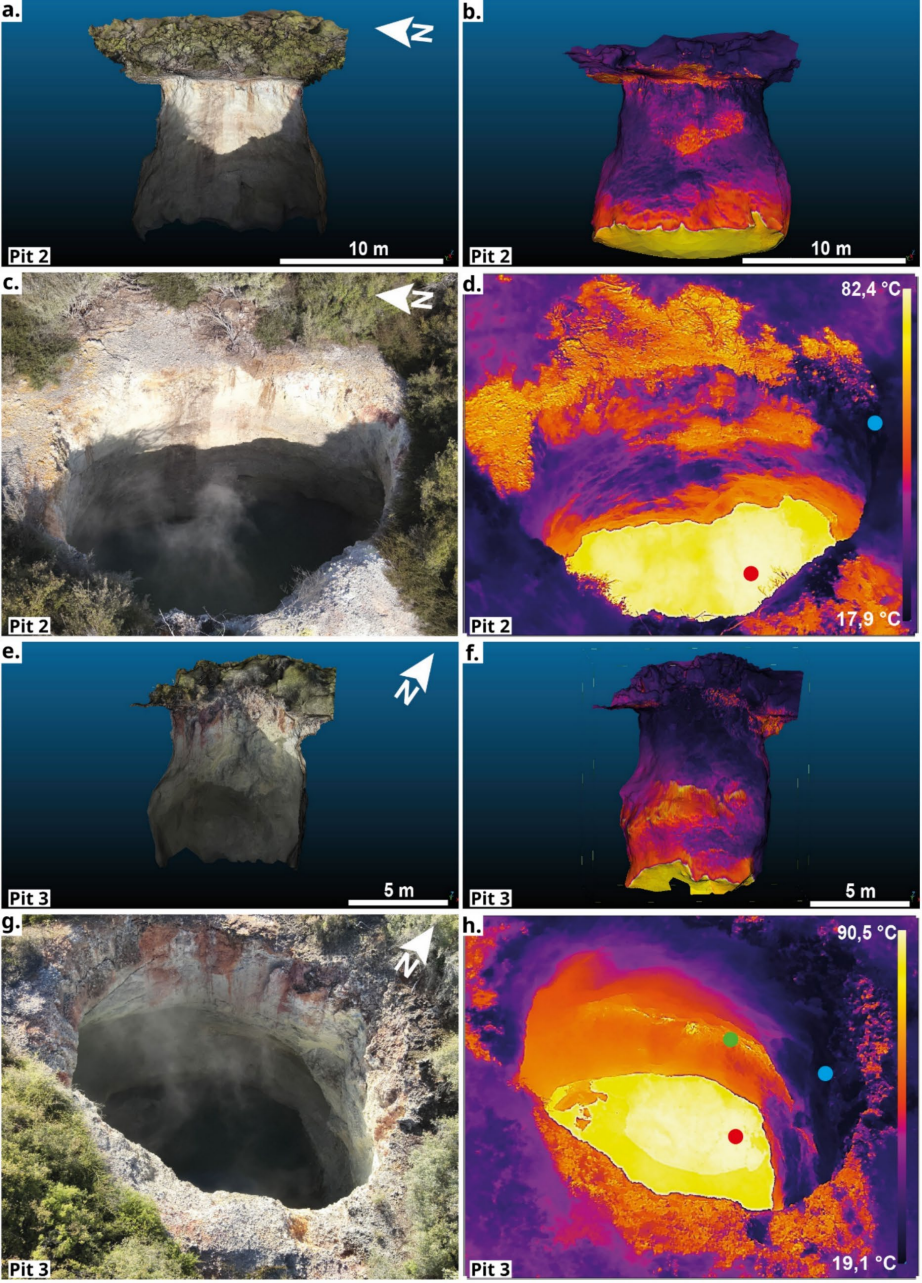
Thermal activity of two selected collapse structures (**a**–**d** pit 2 and **e**–**h** pit 3). **a**, **b**, **e**, **f** represent vertical sections of the visible and thermal 3-D models. **c**, **d**, **g**, **h** represent single visible and thermal images of the collapse structures. Red dots in **d** and **h** indicate the sites with the highest temperatures in the image, and blue dots the sites with the lowest temperatures. The green dot in **h** represents the highest temperature on the wall of the collapse structure

The resulting pit morphology obtained by high-resolution UAS surveys (Figs. 7, and 8) reflects the interplay between collapse processes, hydrothermal alteration intensity, and the duration of postformation modification.

Morphologically, less modified pits display a narrow surface aperture, steep to locally overhanging walls, and a circular to subcircular downward-flaring geometry, with the cavity widening at depth (pits 1, 2, 4, and 5; Figs. 7 and 8). We use these features as relative indicators of limited postformation wall retreat and infilling, rather than as absolute age constraints.

Dimensions vary, with diameters ranging from a few meters to 40 m (e.g., pit 9) and a maximum depth of about 8 m.

Three pits (5, 7, and 9) exhibit irregular, noncircular geometries. This variability likely reflects structural control exerted by underlying volcano-tectonic lineaments and/or structural anisotropy within the host rocks, which may not always be evident at the surface but can govern hydrothermal fluid ascent, creating preferential upflow pathways that influence the spatial development of the pits.

In contrast, more morphologically evolved pits (e.g., pit 9) exhibit broader and more subdued surface depressions. We interpret these geometries as the result of progressive rim retreat, wall slumping, gravitational redistribution of altered material, and possible infilling. These criteria provide a relative geomorphic classification only.

Several pits host water ponds at their bottom (pits 2, 3, 4, 5, 7, and 9; Figs. 7 and 8), with temperatures reaching and locally exceeding 90 °C (max *T* at pit 9: 92.3 °C; min *T* at pit 7: 75.8 °C). Thermal observations reveal spatially heterogeneous temperature distributions within the pits, with discrete up-flow zones located both on the pond floors (pits 2, 3, and 9; Figs. 7 and 8) and along their margins (pits 1, 7, and 3; Figs. 7 and 8), marking sites of focused hydrothermal fluid discharge. Rather than exhibiting uniform thermal conditions, the ponds commonly exhibit convective circulation, reflecting active heat and mass transfer within the hydrothermal system.

No clear spatial pattern was observed in the distribution of the different pit morphologies across the investigated area, suggesting that their development is primarily controlled by localized subsurface conditions, such as structural permeability and hydrothermal fluid flow.

Overall, the temperatures in collapse pits range from 12 to 95 °C. However, local maximum temperatures may be underestimated where hot ground or water surfaces are averaged with surrounding cooler areas within individual thermal pixels (Fig. 7).

## Discussion

### Soil alteration and surficial features

**Fig. 9:**
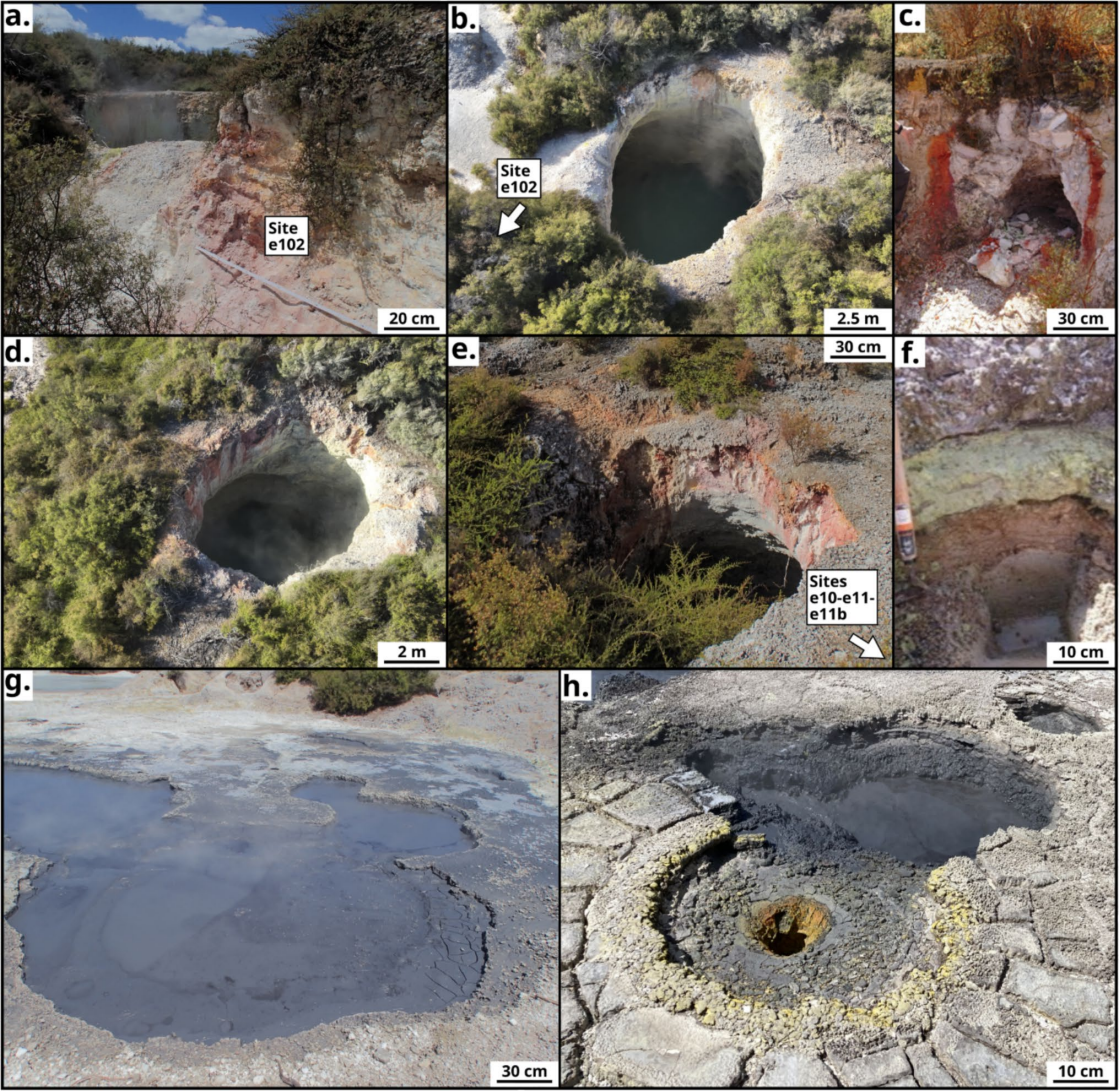
Examples of surficial thermal features and hydrothermal alteration structures in the DOC-thermal area of the Rotokawa geothermal field. **a, b** Highly altered margins and overhanging walls of pit 2 (Figs. 7 and 8) near site e102 that was further analyzed using a UAS survey. **c** Example of an alteration halo and its effect on the surrounding soil layers. **d** Deep collapse pit hosting a bubbling thermal pool (pit 3, Figs. 7 and 8), that was further analyzed with an UAS survey. **e** Collapse structure developed in strongly altered soils near sites e10, e11, and e11b. **f** Example of a sulfur-cemented layer capping more porous soil layers. **g** Boiling mud pool with associated sulfur-encrusted surfaces. **h** Sulfur precipitation and mud-crack textures around a boiling mud pool. These features illustrate the close association between intense alteration, mechanical weakening, and focused surface degassing

Hydrothermal alteration exerts a strong control on the distribution of surficial features and degassing patterns in the thermal area of the Rotokawa geothermal field. Areas of intense alteration coincide with collapse structures, mud pools, fumaroles, sulfur crusts, and hard ground surfaces, reflecting the interaction between steam-heated fluids, meteoric water, and the pumice-rich soils that dominate the upper stratigraphy (Fig. 9a–f).

High CO_2_ fluxes reported by earlier studies (Bloomberg et al. 2014; Yang et al. 2024) occur preferentially within these altered zones. Hydrothermal alteration modifies soil properties through dissolution, leaching, cementation, and mechanical weakening—processes documented in geothermal systems such as Kambalny (Russia), Whakaari/White Island (New Zealand), Campi Flegrei (Italy), Vulcano (Italy), and the Valley of Desolation (Dominica) (Frolova et al. 2014, 2016, 2019; Heap et al. 2015; Mayer et al. 2017; Montanaro et al. 2017; Harris and Pailot-Bonnétat 2024). Dissolution is expected to increase porosity and permeability while reducing grain cohesion, producing friable soils that may be prone to subsidence and collapse (e.g.,Schöpa et al. 2011; Rott et al. 2019; Frolova et al. 2021). In contrast, silica, sulfur, and clay precipitation reduce permeability and may strengthen shallow horizons, generating hard grounds or sealed layers (e.g. Piochi et al. 2015; Ricci et al. 2015; Semenkov et al. 2021; Montanaro et al. 2023).

Our new dataset includes locally higher permeability values than those reported by Montanaro et al. (2023), particularly within altered T3 soils. Given the limited spatial coverage of the surveys and the strong meter- or even decimeter-scale heterogeneity of steam-heated soils, this likely reflects the direct sampling of highly altered, friable T3 domains and the intrinsic variability of T3 soils rather than a systematic field-wide increase. These higher permeability, friable zones coincide with white-to-red weakened T3 exposed along collapse margins (Figs. 6a and 9a, b, d, and e), where dissolution and grain disaggregation may contribute to increased porosity, consistent with field-scale observations of weakened material, and are characterized by an enrichment in particles measuring 125–16 μm. In contrast, sulfur-cemented surfaces near fumaroles, mud pools, and excavated areas (Fig. 9a and e) exhibit markedly lower permeability, consistent with localized shallow sealing inferred from surface mineral crusts and low-permeability horizons described in this and previous studies (Montanaro et al. 2023).

These locally permeable altered T3 domains occur adjacent to clay-rich C1–C2 horizons, whose hydraulic behavior is considerably more heterogeneous than implied by their soil type classification. Despite forming sharp contacts with underlying T3, the C1–C2 horizons display permeabilities spanning the full range of measured values (1.2 × 10^–13^ to > 6.5 × 10^–13^ m^2^), reflecting the strong sensitivity of clay-rich materials to saturation state, microstructural arrangement, and alteration intensity, as documented for plastic soils (Chapuis 2012). Their uniformly fine grain size (< 4 µm) therefore provides little predictive value, since permeability in such media is controlled primarily by clay fabric, plasticity, and pore connectivity rather than particle size alone (Heap et al. 2015; Díaz-Curiel et al. 2022). Field measurements show that in some sites (e.g., e14 and e108–e12) the C1–C2 horizons can be as permeable as T3, whereas in others (e11 and e102) they act as semiconfining units. This behavior is best explained by variations in plasticity, degree of saturation, and alteration-related structural continuity rather than intrinsic permeability contrasts. Horizontal and vertical permeability measurements reveal no systematic anisotropy at the scale and configuration of our field measurements, indicating that local alteration and microtexture might exert stronger controls on shallow permeability than layer orientation.

### Shallow fluid pathways through surficial soils

Diffuse soil CO_2_ flux measurements are widely used to infer degassing activity and reservoir conditions of active hydrothermal systems, yet they only capture gas escape at the ground surface and do not resolve how shallow stratigraphy modulates subsurface migration (Bloomberg et al. 2014; Maier and Schack-Kirchner 2014; Harvey et al. 2017; Yang et al. 2024). To address this limitation, we combined horizontal and vertical interstitial soil gas concentration profiles with petrophysical measurements to directly characterize fluid pathways within the upper decimeters of the Rotokawa soil column.

The typical pattern of CO₂ concentrations in vertical profiles in areas of diffuse degassing shows increasing concentrations with depth, reflecting gas generation or upward migration from deeper sources (e.g., Camarda et al. 2006). This baseline pattern provides the reference framework for interpreting local deviations produced by lithological contrasts and the presence of collapse structures. Permeable pumice-rich T3 and T4 layers consistently act as primary gas pathways, in accordance with their open textures and high porosity (Figs. 3–6). In contrast, clay-rich C1 horizons locally restrict vertical gas flow, producing pronounced CO_2_ peaks at T3–C1 contacts (e.g., at sites e2, e14, and e11; Figs. 3, 4, and 5). These physical property contrasts promote lateral diversion of gas along clay margins or fractures (e.g., e2 in Fig. 3), a behavior consistent with observations in other altered geothermal soils (Camarda et al. 2006, 2017; Ricci et al. 2015).

At site e2, CO_2_ maxima align with the T3-C1 interface, and vertical profiles show near-complete suppression of upward gas flow where the clays are thickest (Fig. 3). Lateral reactivation of ascent along the clay margins highlights the sensitivity of shallow gas flow to small permeability variations, similar to patterns that have been described in steam-heated terrains across several geothermal areas in New Zealand (Rissmann et al. 2012; Bloomberg et al. 2014). Fluids exhibit a different behavior in the proximity of collapse structures. At site e102, CO_2_, CH_4_, and H_2_O vapor concentrations increase toward permeable altered T3 but decrease sharply at the collapse boundary (Fig. 6), suggesting gas capture, redistribution, or lateral migration along the collapse structure.

UAS-derived 3-D visible and thermal models of selected pits (Fig. 8) allowed us to characterize the geometry and thermal anomalies within the collapse structures. We selected two of the most representative pits to elucidate contrasting thermal and fluid-flow expressions within collapse structures. The small surface opening size, along with the vertical walls and downwardly flared geometry of the two collapse structures, is consistent with limited post-formation wall retreat and infilling. In Fig. 8a–d, there is no clear evidence of wall-hosted endogenous fluids discharge. The highest temperature (82.4°C) is recorded at the surface of the liquid pool within the pit, indicating that the primary fluid contribution originates from below. The thermal anomaly visible in the upper part of the structure is instead mainly due to solar activity, as evidenced by the geometry of the shadows in Fig. 8a, c. By contrast, clear thermal anomalies are visible both on the surface of the boiling pool (90,5°C), highlighted by convective dynamics, and in the middle of the wall (67.5°C; Fig. 8f, h).

The integration of field measurements with UAS-derived visible and thermal models suggests that collapse pit morphology and thermal structure are influenced by the same layer-scale properties that govern shallow gas migration. The petrophysical and gas-profile data indicate that altered pumice-rich T3 and T4 horizons can remain relatively permeable while becoming mechanically weakened. Such layers may favor focused gas ascent, localized alteration, and progressive weakening of pit margins. By contrast, clay-rich C1–C2 horizons and locally sulfur- or silica-cemented layers may behave as transient low-permeability barriers, limiting vertical gas escape and promoting lateral migration of CO_2_-rich fluids. Where these horizons are exposed or breached along collapse margins, gas and heat can be focused along pit walls or structural discontinuities, potentially explaining the wall-hosted thermal anomalies observed in some pit thermal models. Pit-bottom or pond-floor anomalies, on the other hand, are more consistent with direct vertical upflow beneath the collapse structures. Therefore, the UAS data complement the in-situ measurements by showing how shallow permeability contrasts, alteration intensity, and mechanical weakening are expressed at the scale of collapse-pit morphology and thermal heterogeneity.

**Fig. 10:**
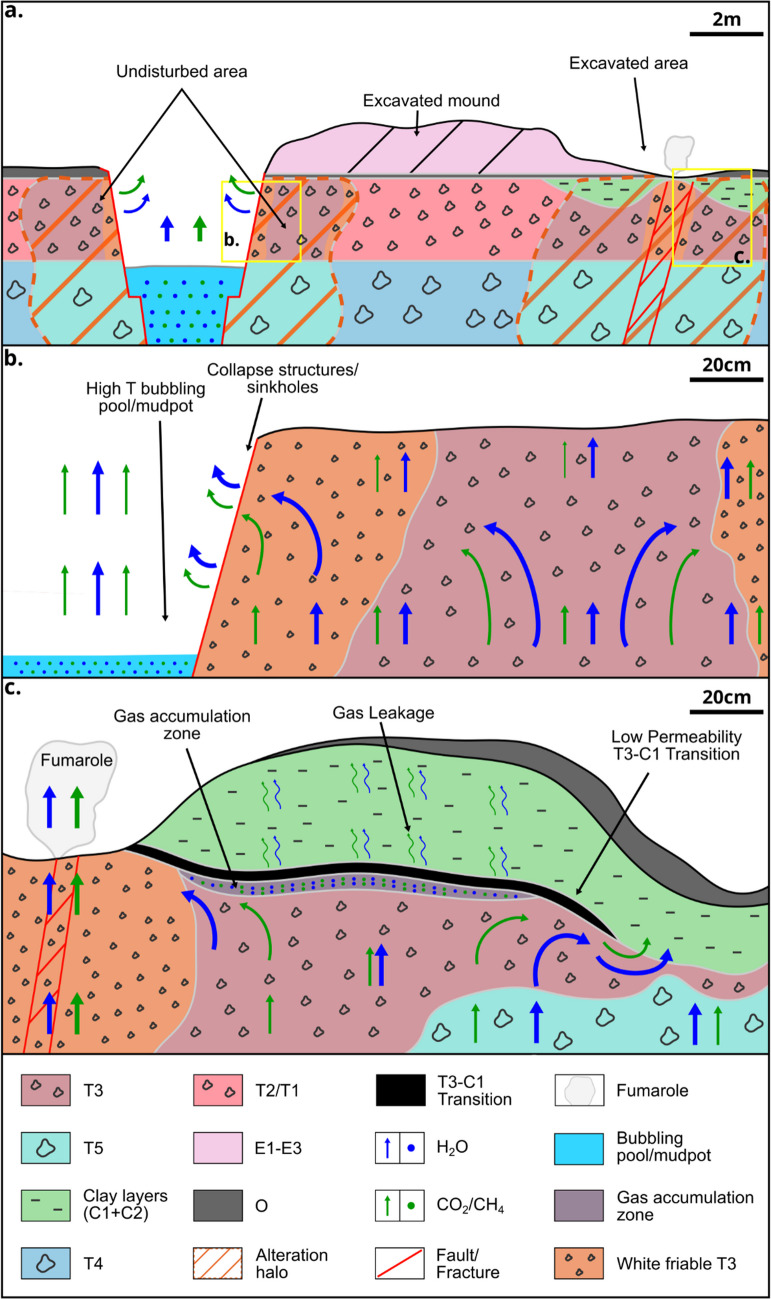
Conceptual model summarizing the controls of soil stratigraphy, hydrothermal alteration, and collapse structures on shallow fluid flow in the Rotokawa DOC thermal area. Lithology symbol size reflects representative grain size. **a** Overview of the main surficial soil types, including pumice-rich Taupō Pumice Formation soils (T1–T5), clay-rich horizons (C1–C2), excavated reworked materials (E1–E3), and variegated silty to sandy layers with organic matter (O), together with associated surface expressions such as fumaroles, mud pools, alteration halos, and collapse depressions. **b** Interaction between alteration halos and collapse structures, where increased permeability within altered T3 promotes vertical and lateral gas flow. Collapse structures act as preferential permeable conduits. **c** Layered-sequence scenario dominated by soil-type contrasts, where permeable T3 favors upward gas ascent, while overlying clay layers (C1–C2) act as semiconfining horizons, promoting CO_₂_ and CH_₄_ accumulation, lateral migration, and shallow gas–steam decoupling through steam condensation. Soil types, gas flow direction, and thermal features are explained in the legend. Orange areas in **b** and **c** indicate the most intensely altered portions of T3. The horizontal scale shown in each panel applies equally to the vertical axis

The interpretations of our new combined data are summarized in a conceptual model presented in Fig. 10. The shallow stratigraphy of the Rotokawa thermal area comprises pumice-rich Taupō Pumice Formation soils (T1–T5) in undisturbed areas and excavated reworked materials (E1–E3) overlying clay-rich contact layers (C1–C2). Across both settings, CO_2_-rich fluids degassing produce fumaroles, mud pools, alteration halos, and collapse depressions (Fig. 10a). In zones where alteration halos intersect collapse structures and fluid flow is present, we observe that both alteration halos and collapse structures control fluid flow patterns. Alteration halos and collapse structures strongly reorganize fluid pathways. Increased permeability in altered T3 enhances vertical and lateral gas flow, while collapse margins act as permeable conduits or sinks that focus or capture rising gases, generating localized degassing and thermal anomalies (Fig. 10b). In layered sequences, contrasts in soil type dominate fluid flow. Permeable T3 permits upward gas flow, but overlying C1–C2 horizons act as semi-confining layers that suppress vertical ascent, causing CO_2_ to accumulate and migrate laterally along the T3–C1 interface. Steam condensation beneath the clay promotes shallow sealing and gas–steam decoupling, reinforcing lateral redistribution before upward escape occurs at discontinuities (Fig. 10c).

### Broader implications: permeability evolution and long-term degassing trends

Self-sealing can exert an important time-dependent influence on shallow permeability in steam-heated terrains. Steam condensation beneath clay-rich horizons and around fumaroles and mud pools promotes rapid precipitation of sulfur, amorphous silica, and hydrothermal clays, forming thin, transient low-permeability skins (Rodgers et al. 2002; Harris et al. 2009; Sillitoe 2015; Jones and Detwiler 2016; Madonia et al. 2016). Similar sealing textures observed in steam-heated systems at Mutnovsky (Russia), Kambalny (Russia), and Whakaari/White Island (New Zealand) demonstrate that these horizons can develop and decay quickly in response to fluctuations in temperature, humidity, and gas flux (Frolova et al. 2014; Christenson et al. 2017; Heap et al. 2017; Sergeeva et al. 2019; Kiryukhin et al. 2020). As sealing develops, steam condenses and reinforces mineral deposition, while noncondensable CO_2_ may accumulate below or adjacent to semiconfining horizons and be diverted laterally towards a permeable discontinuity. This gas–steam decoupling mechanism (Henley and Ellis 1983; Peiffer et al. 2018; Fowler et al. 2019; Taussi et al. 2019) is consistent with the strong CO_2_ gradients observed across C1–C2 interfaces and the localized CO_2_ depletion near collapse margins at Rotokawa. Crack development and elutriation pipes can eventually cut through low-permeability seals, allowing gases to locally reach the surface (Cody 2003; Heap et al. 2015; Montanaro et al. 2017, 2023).

Shallow processes intersect with longer-term changes in the degassing system. Diffuse CO_2_ emissions at Rotokawa have declined from ~ 441 t d^−1^ in 2011 to ~ 345 t d^−1^ in 2023 (Bloomberg et al. 2014; Yang et al. 2024), paralleling the reduction in CO_2_-equivalent discharge from nearby power stations. Changes in deep CO_2_ supply related to reservoir-pressure evolution remain a plausible first-order explanation, consistent with trends at Ohaaki, Wairakei, and other exploited fields within the TVZ (Giggenbach 1995; Glover and Mroczek 1995; Rissmann et al. 2012). In addition, progressive sealing and re-focusing of gas into structural or permeable soil “conduits” may also reduce the areal footprint of diffuse emissions without necessarily requiring a proportional reduction in the deep flux, as observed at several stratovolcanoes and geothermal fields (Viveiros et al. 2010; Ricci et al. 2015; Epiard et al. 2017; Harvey et al. 2018; Frolova et al. 2019; Chiodini et al. 2021; Müller et al. 2024; Hendriawan et al. 2025). This interpretation should therefore be regarded as complementary to reservoir-scale controls, rather than as an alternative explanation for the reported emission decrease. Environmental and methodological variability (Lewicki et al. 2005; Viveiros et al. 2009; Hernández et al. 2012; Werner et al. 2019) further complicates inter-decadal comparisons. To resolve the relative impacts of near-surface soil processes on overall degassing, longitudinal monitoring of soil properties, gas fluxes and structural changes will be needed. Such monitoring is essential for improving hazard assessment and exploitation strategies and should account for how surficial soil lithologies and hydrothermal alteration redistribute gas flow pathways.

## Conclusions

This multiparametric and multidisciplinary study aims to unravel the complex interplay between surficial fluid flow patterns, thermal and geological features, anthropogenic activity and hydrothermal alteration. We conclude the following:

- The shallow soil profile at the Rotokawa geothermal field exerts a primary control on gas migration and degassing patterns.
- Pyroclastic deposits of the pumice-rich Taupō Pumice Formation form the main permeable framework for CO_2_ ascent, whereas clay-rich horizons and reworked mining deposits can locally act as semiconfining layers, with gas concentrating at lithological boundaries and being diverted laterally.
- Sharp gas depletions near collapse margins indicate that meter-scale contrasts in lithology, alteration and structure govern how gases move through the upper decimeters of the system.
- Alteration halos and collapse structures control shallow gas migration pathways by enhancing permeability within weakened pyroclastic domains and by locally focusing or capturing gas along collapse margins. CO_2_, CH_4_, and H_2_O vapor concentrations increase towards highly altered horizons but decrease at collapse margins, indicating lateral or downward redistribution or capture.
- UAS-derived 3-D contrasting thermal patterns, with morphologically less modified collapse structures displaying the highest temperatures at the bottom of the pits, while larger collapse or more morphologically evolved collapse structures show clear thermal anomalies both on the surface of the boiling pool and in the middle of the pit wall.
- Progressive shallow sealing can redirect gas into fewer, more focused conduits, with soil lithologies and hydrothermal alteration redistributing preferential gas migration pathways. These processes may contribute to apparent decreases of gas emissions at the geothermal field scale, alongside reservoir processes, environmental variability and methodological uncertainty.

Together, our results show that shallow soils are not passive boundaries but dynamic, evolving components of the geothermal system, and that robust interpretation of long-term degassing trends requires joint consideration of reservoir processes, near-surface permeability evolution and environmental forcing, with direct implications for monitoring strategies, hazard assessment and conceptual models of steam-heated terrains.

## Supplementary Information

Below is the link to the electronic supplementary material.

ESM 1 (XLSX.11.7 KB)

ESM 2 (DOCX 6.34 MB)

## Data Availability

All the data used for the present study are provided in the main text, figures, and the supplementary table.

## References

[CR1] Addison S, Winick J, Mountain B, Siega F (2015) Rotokawa reservoir tracer test history. Proc. NZ Geothermal Workshop

[CR2] Bardsley C, Williams K (2017) The dawn of Rotokawa: lines on the land (map series 2). In, https://mrpmaps.maps.arcgis.com/apps/MapSeries/index.htmlappid=2595f98e263d4f4db895bbe0694e2938

[CR3] Bettersizer (2022) Bettersizer S3 Plus. User manual. https://bettersizeinstruments.com/uploads/file/bettersizer-s3-plus-product-brochure-english.pdf

[CR4] Bini G, Chiodini G, Ricci T, Sciarra A, Caliro S, Mortensen AK, Martini M, Mitchell A, Santi A, Costa A (2024) Soil CO_2_ emission and stable isotopes (δ13C, δ18O) of CO_2_ and calcites reveal the fluid origin and thermal energy in the supercritical geothermal system of Krafla, Iceland. J Volcanol Geotherm Res 447:108032, 108032

[CR5] Bloomberg S, Werner C, Rissmann C, Mazot A, Horton T, Gravley D, Kennedy B, Oze C (2014) Soil CO_2_ emissions as a proxy for heat and mass flow assessment, Taupō Volcanic Zone, New Zealand. Geochem Geophys Geosyst 15(12):4885–4904

[CR6] Brooks-Clarke IA (2021) Mineralogical insights into hydrothermal eruption conditions at Rotokawa Geothermal Field, New Zealand. In: ResearchSpace@ Auckland,

[CR7] Browne P (1978) Hydrothermal alteration in active geothermal fields. In: Annual review of earth and planetary sciences. Volume 6.(A78–38764 16–42) Palo Alto, Calif., Annual Reviews, Inc., 1978, p. 229–250. 6:229–250

[CR9] Camarda M, Gurrieri S, Valenza M (2006) CO2 flux measurements in volcanic areas using the dynamic concentration method: influence of soil permeability. Journal of Geophysical Research: Solid Earth 111(B5)

[CR10] Camarda M, Gurrieri S, Valenza M (2009) Effects of soil gas permeability and recirculation flux on soil CO2 flux measurements performed using a closed dynamic accumulation chamber. Chem Geol 265(3–4):387–393

[CR11] Camarda M, Prano V, Cappuzzo S, Gurrieri S, Valenza M (2017) Temporal variations in air permeability and soil CO2 flux in volcanic ash soils (island of Vulcano, Italy). Geochem Geophys Geosyst 18(8):3241–3253

[CR12] Cardellini C, Chiodini G, Frondini F, Avino R, Bagnato E, Caliro S, Lelli M, Rosiello A (2017) Monitoring diffuse volcanic degassing during volcanic unrests: the case of Campi Flegrei (Italy). Sci Rep 7(1):6757 28754925 10.1038/s41598-017-06941-2 PMC5533770

[CR13] Chambefort I (2021) Sulfur in New Zealand geothermal systems: insights from stable isotope and trace element analyses of anhydrite from Rotokawa and Ngatamariki geothermal fields, Taupo Volcanic Zone. NZ J Geol Geophys 64:372–388

[CR14] Chapuis RP (2012) Predicting the saturated hydraulic conductivity of soils: a review. Bull Eng Geol Env 71(3):401–434

[CR15] Chiodini G, Cioni R, Guidi M, Raco B, Marini L (1998) Soil CO2 flux measurements in volcanic and geothermal areas. Appl Geochem 13(5):543–552

[CR16] Chiodini G, Caliro S, Avino R, Bini G, Giudicepietro F, De Cesare W, Ricciolino P, Aiuppa A, Cardellini C, Petrillo Z (2021) Hydrothermal pressure-temperature control on CO2 emissions and seismicity at Campi Flegrei (Italy). J Volcanol Geoth Res 414:107245

[CR17] Christenson B, White S, Britten K, Scott B (2017) Hydrological evolution and chemical structure of a hyper-acidic spring-lake system on Whakaari/White Island, NZ. J Volcanol Geoth Res 346:180–211

[CR18] Civico R, Ricci T, Scarlato P, Taddeucci J, Andronico D, Del Bello E, D’Auria L, Hernandez PA, Perez NM (2022) High-resolution digital surface model of the 2021 eruption deposit of Cumbre Vieja volcano, La Palma. Spain Sci Data 9(1):435 35902600 10.1038/s41597-022-01551-8 PMC9334277

[CR19] Civico R, Ricci T, Cecili A, Scarlato P (2024) High-resolution topography reveals morphological changes of Stromboli volcano following the July 2024 eruption. Sci Data 11(1):1219 39532938 10.1038/s41597-024-04098-y PMC11557571

[CR20] Cody AD (2003) Geology, history and stratigraphy of hydrothermal eruptions in the rotorua geothermal field. Doctoral dissertation, University of Waikato

[CR21] Collar R (1985) Hydrothermal eruptions in the Rotokawa geothermal system, Taupo Volcanic Zone. Geothermal Institute, University of Auckland, New Zealand

[CR22] De Landro G, Serlenga V, Russo G, Amoroso O, Festa G, Bruno PP, Gresse M, Vandemeulebrouck J, Zollo A (2017) 3D ultra-high resolution seismic imaging of shallow Solfatara crater in Campi Flegrei (Italy): new insights on deep hydrothermal fluid circulation processes. Sci Rep 7(1):3412 28611382 10.1038/s41598-017-03604-0 PMC5469761

[CR23] Díaz-Curiel J, Miguel MJ, Biosca B, Arévalo-Lomas L (2022) New granulometric expressions for estimating permeability of granular drainages. Bull Eng Geol Env 81(10):397

[CR24] Epiard M, Avard G, De Moor JM, Martínez Cruz M, Barrantes Castillo G, Bakkar H (2017) Relationship between diffuse CO2 degassing and volcanic activity. Case study of the Poás, Irazú, and Turrialba Volcanoes, Costa Rica. Front Earth Sci 5:71, 71

[CR25] Evans WC, Sorey M, Kennedy B, Stonestrom DA, Rogie J, Shuster D (2001) High CO2 emissions through porous media: transport mechanisms and implications for flux measurement and fractionation. Chem Geol 177(1–2):15–29

[CR26] Farquhar G (2001) Guideline for hand held shear vane test. New Zealand Geotechnical Society

[CR27] Fowler AP, Tan C, Cino C, Scheuermann P, Volk MW, Shanks WP III, Seyfried WE Jr (2019) Vapor-driven sublacustrine vents in Yellowstone Lake, Wyoming, USA. Geology 47(3):223–226

[CR28] Frolova J, Ladygin V, Rychagov SN, Zukhubaya D (2014) Effects of hydrothermal alterations on physical and mechanical properties of rocks in the Kuril-Kamchatka island arc. Eng Geol 183:80–95

[CR29] Frolova YV, Rychagov SN, Ladygin VM, Luchko MV, Chernov MS, Boikova IA (2016) Variation in the physical and mechanical properties of rocks: the North Paramushir hydrothermal magmatic system, Kuril Islands. J Volcanol Seismolog 10(3):170–187

[CR30] Frolova J, Chernov M, Rychagov S, Kuznetsov R, Surovtseva K (2019) Alteration of volcanic rocks and changes in physical-mechanical properties on the South-Kambalny thermal field (South Kamchatka). E3S Web of Conferences 98:08002

[CR31] Frolova JV, Chernov MS, Rychagov SN, Ladygin VM, Sokolov VN, Kuznetsov RA (2021) The influence of hydrothermal argillization on the physical and mechanical properties of tuffaceous rocks: a case study from the Upper Pauzhetsky thermal field, Kamchatka. Bull Eng Geol Environ 80(2):1635–1651

[CR32] Gaudin D, Ricci T, Finizola A, Delcher E, Alparone S, Barde-Cabusson S, Brothelande É, Di Gangi F, Gambino S, Inguaggiato S (2017) Heat flux-based strategies for the thermal monitoring of sub-fumarolic areas: examples from Vulcano and La Soufrière de Guadeloupe. J Volcanol Geotherm Res 343:122–134

[CR33] Giggenbach WF (1995) Variations in the chemical and isotopic composition of fluids discharged from the Taupo Volcanic Zone, New Zealand. J Volcanol Geotherm Res 68(1–3):89–116

[CR34] Glover R, Mroczek E (1995) Impact of fluid chemistry on power development at Rotokawa, New Zealand. Proceedings of World Geothermal Congress

[CR35] Harris AJ, Pailot-Bonnétat S (2024) Inversion of heat loss to obtain conductivity, density, and permeability at bottom-heated surfaces: the case of the hydrothermal system at Vulcano between 2019 and 2023. Bull Volcanol 86(6):55

[CR36] Harris AJ, Lodato L, Dehn J, Spampinato L (2009) Thermal characterization of the Vulcano fumarole field. Bull Volcanol 71(4):441–458

[CR37] Harvey M, Rowland J, Chiodini G, Rissmann C, Bloomberg S, Fridriksson T, Oladottir A (2017) CO2 flux geothermometer for geothermal exploration. Geochim Cosmochim Acta 213:1–16

[CR38] Harvey M, Chavez G, Delgado M (2018) CO2 flux surveys for geothermal exploration in arid environments. 43rd Workshop on Geothermal Reservoir Engineering. Stanford, California

[CR40] Heap MJ, Kennedy BM, Pernin N, Jacquemard L, Baud P, Farquharson JI, Scheu B, Lavallée Y, Gilg HA, Letham-Brake M, Mayer K, Jolly AD, Reuschlé T, Dingwell DB (2015) Mechanical behaviour and failure modes in the Whakaari (White Island volcano) hydrothermal system, New Zealand. J Volcanol Geoth Res 295:26–42

[CR42] Heap MJ, Kennedy BM, Farquharson JI, Ashworth J, Mayer K, Letham-Brake M, Reuschlé T, Gilg HA, Scheu B, Lavallée Y, Siratovich P, Cole J, Jolly AD, Baud P, Dingwell DB (2017) A multidisciplinary approach to quantify the permeability of the Whakaari/White Island volcanic hydrothermal system (Taupo Volcanic Zone, New Zealand). J Volcanol Geoth Res 332:88–108

[CR43] Hedenquist J, Mroczek E, Giggenbach W (1988) Geochemistry of the Rotokawa geothermal system: summary of data, interpretation and appraisal for energy development. Chemistry Division DSIR Technical Note 88(6)

[CR44] Hendriawan R, Kaya E, Zarrouk SJ, Luketina K, Bromley C (2025) Shallow geothermal subsurface temperature contour mapping aided by numerical reservoir modelling; Taupō. New Zealand. Geothermics 131:103380, 103380

[CR45] Henley RW, Ellis AJ (1983) Geothermal systems ancient and modern: a geochemical review. Earth Sci Rev 19(1):1–50

[CR46] Hernández PA, Padilla G, Padrón E, Pérez NM, Calvo D, Nolasco D, Melián G, Barrancos J, Dionis S, Rodríguez F (2012) Analysis of long-and short-term temporal variations of the diffuse CO2 emission from Timanfaya volcano, Lanzarote. Canary Islands Applied Geochemistry 27(12):2486–2499

[CR47] Hopp C, Sewell S, Mroczek S, Savage M, Townend J (2020) Seismic response to evolving injection at the Rotokawa geothermal field. New Zealand Geothermics 85:101750

[CR48] Hughes EC, Mazot A, Kilgour G, Asher C, Michelini M, Britten K, Chardot L, Feisel Y, Werner C (2019) Understanding degassing pathways along the 1886 tarawera (New Zealand) volcanic fissure by combining soil and lake CO2 fluxes. Front Earth Sci 7:474454

[CR49] Isaia R, Vitale S, Di Giuseppe MG, Iannuzzi E, D’Assisi Tramparulo F, Troiano A (2015) Stratigraphy, structure, and volcano-tectonic evolution of Solfatara maar-diatreme (Campi Flegrei, Italy). Bulletin 127(9–10):1485–1504

[CR50] Jones TA, Detwiler RL (2016) Fracture sealing by mineral precipitation: the role of small-scale mineral heterogeneity. Geophys Res Lett 43(14):7564–7571

[CR51] Jury A (1984) The Rotokawa sulphur deposits. In: Proceedings 18th annual australasian institute of mining and metallurgy New Zealand branch conference. pp 192–205

[CR52] Kiryukhin A, Rychkova T, Sergeeva A (2020) Simulating the conditions of generation for permeable geyser channels in areas of acid volcanism. J Volcanol Seismolog 14(2):71–82

[CR53] Klein A, Jessop DE, Donnadieu F, Pierre J, Moretti R (2024) Dome permeability and fluid circulation at La Soufrière de Guadeloupe implied from soil CO2 degassing, thermal flux and self-potential. Bull Volcanol 86(4):26

[CR54] Krupp R, Seward T (1987) The Rotokawa geothermal system, New Zealand; an active epithermal gold-depositing environment. Econ Geol 82(5):1109–1129

[CR55] Lewicki JL, Bergfeld D, Cardellini C, Chiodini G, Granieri D, Varley N, Werner C (2005) Comparative soil CO2 flux measurements and geostatistical estimation methods on Masaya volcano. Nicaragua Bulletin of Volcanology 68(1):76–90

[CR56] Madonia P, Cangemi M, Costa M, Madonia I (2016) Mapping fumarolic fields in volcanic areas: a methodological approach based on the case study of La Fossa cone, Vulcano island (Italy). J Volcanol Geotherm Res 324:1–7

[CR57] Maier M, Schack-Kirchner H (2014) Using the gradient method to determine soil gas flux: a review. Agric for Meteorol 192:78–95

[CR58] Mayer K, Scheu B, Yilmaz TI, Montanaro C, Albert Gilg H, Rott S, Joseph EP, Dingwell DB (2017) Phreatic activity and hydrothermal alteration in the Valley of Desolation, Dominica. Lesser Antilles. Bull Volcanol 79(12):1–19

[CR59] McNamara DD, Sewell S, Buscarlet E, Wallis IC (2016) A review of the Rotokawa geothermal field, New Zealand. Geothermics 59:281–293

[CR60] Mir BA (2021) Manual of geotechnical laboratory soil testing. CRC Press

[CR61] Montanaro C, Mayer K, Isaia R, Gresse M, Scheu B, Yilmaz TI, Vandemeulebrouck J, Ricci T, Dingwell DB (2017) Hydrothermal activity and subsoil complexity: implication for degassing processes at Solfatara crater, Campi Flegrei caldera. Bulletin of Volcanology 79(12)

[CR62] Montanaro C, Ray L, Cronin SJ, Calibugan A, Rott S, Bardsley C, Scheu B (2023) Linking top and subsoil types, alteration and degassing processes at Rotokawa geothermal field, New Zealand. Frontiers in Earth Science 10

[CR63] Mousavi F, Abdi E, Ghalandarayeshi S, Page-Dumroese DS (2021) Modeling unconfined compressive strength of fine-grained soils: application of pocket penetrometer for predicting soil strength. CATENA 196:104890

[CR64] Müller D, Walter TR, Troll VR, Stammeier J, Karlsson A, de Paolo E, Pisciotta AF, Zimmer M, De Jarnatt B (2024) Anatomy of a fumarole field: drone remote-sensing and petrological approaches reveal the degassing and alteration structure at La Fossa cone, Vulcano. Italy Solid Earth 15(9):1155–1184

[CR65] Peiffer L, Carrasco-Núñez G, Mazot A, Villanueva-Estrada RE, Inguaggiato C, Romero RB, Miller RR, Rojas JH (2018) Soil degassing at the Los Humeros geothermal field (Mexico). J Volcanol Geotherm Res 356:163–174

[CR66] Piochi M, Mormone A, Balassone G, Strauss H, Troise C, De Natale G (2015) Native sulfur, sulfates and sulfides from the active Campi Flegrei volcano (southern Italy): genetic environments and degassing dynamics revealed by mineralogy and isotope geochemistry. J Volcanol Geoth Res 304:180–193

[CR67] Pirajno F (2020) Subaerial hot springs and near-surface hydrothermal mineral systems past and present, and possible extraterrestrial analogues. Geosci Front 11(5):1549–1569

[CR68] Pola A, Crosta GB, Fusi N, Castellanza R (2014) General characterization of the mechanical behaviour of different volcanic rocks with respect to alteration. Eng Geol 169:1–13

[CR69] Renaut RW, Jones B (2011) Hydrothermal environments, terrestrial. Encyclopedia of geobiology. Springer, pp 467–479

[CR70] Ricci T, Finizola A, Barde-Cabusson S, Delcher E, Alparone S, Gambino S, Milluzzo V (2015) Hydrothermal fluid flow disruptions evidenced by subsurface changes in heat transfer modality: the La Fossa cone of Vulcano (Italy) case study. Geology 43(11):959–962

[CR71] Risk G (2000) Electrical resistivity surveys of the Rotokawa geothermal field, New Zealand. In: Proceedings of the 22nd New Zealand Geothermal Workshop. University of Auckland Auckland, pp 121–126

[CR72] Rissmann C, Christenson B, Werner C, Leybourne M, Cole J, Gravley D (2012) Surface heat flow and CO2 emissions within the Ohaaki hydrothermal field, Taupo Volcanic Zone. New Zealand. Appl Geochem 27(1):223–239

[CR73] Rodgers K, Cook K, Browne P, Campbell K (2002) The mineralogy, texture and significance of silica derived from alteration by steam condensate in three New Zealand geothermal fields. Clay Miner 37(2):299–322

[CR74] Rott S, Scheu B, Montanaro C, Mayer K, Joseph EP, Dingwell DB (2019) Hydrothermal eruptions at unstable crater lakes: insights from the Boiling Lake, Dominica, Lesser Antilles. J Volcanol Geoth Res 381:101–118

[CR75] Rowland J, Sibson R (2004) Structural controls on hydrothermal flow in a segmented rift system, Taupo Volcanic Zone. New Zealand Geofluids 4(4):259–283

[CR76] Rowland JV, Simmons SF (2012) Hydrologic, magmatic, and tectonic controls on hydrothermal flow, Taupo Volcanic Zone, New Zealand: implications for the formation of epithermal vein deposits. Econ Geol 107(3):427–457

[CR77] Schöpa A, Pantaleo M, Walter TR (2011) Scale-dependent location of hydrothermal vents: stress field models and infrared field observations on the Fossa Cone, Vulcano Island, Italy. J Volcanol Geoth Res 203(3–4):133–145

[CR78] Semenkov IN, Klink GV, Lebedeva MP, Krupskaya VV, Chernov MS, Dorzhieva OV, Kazinskiy MT, Sokolov VN, Zavadskaya AV (2021) The variability of soils and vegetation of hydrothermal fields in the Valley of Geysers at Kamchatka Peninsula. Sci Rep 11(1):11077 34040134 10.1038/s41598-021-90712-7 PMC8154911

[CR79] Sergeeva A, Denisov D, Nazarova M (2019) Clay mineral assemblages in recent thermal anomalies of Southern Kamchatka. Russ Geol Geophys 60(11):1267–1277

[CR80] Sewell S, Cumming W, Azwar L, Bardsley C (2012) Integrated MT and natural state temperature interpretation for a conceptual model supporting reservoir numerical modelling and well targeting at the Rotokawa Geothermal Field, New Zealand. In: Proceedings of the thirty-seventh workshop on geothermal reservoir engineering. Stanford University, Stanford California

[CR81] Sewell SM, Cumming W, Bardsley CJ, Winick J, Quinao J, Wallis IC, Sherburn S, Bourguignon S (2015) Interpretation of microseismicity at the Rotokawa Geothermal Field, 2008 to 2012. Interpretation 19:25

[CR82] Sillitoe RH (2015) Epithermal paleosurfaces. Miner Deposita 50(7):767–793

[CR83] Simpson M, Morales A, Chambefort I, Alcaraz S, Moribe S, Milicich S, Calibugan A, Grove T (2021) Hydrothermal minerals and hydrologic evolution of the Rotokawa geothermal system, New Zealand. In: New Zealand geothermal workshop 2021. University of Auckland Auckland, New Zealand, p 8

[CR84] Sinclair B, Kear D (1989) Lake Rotokawa sulphur deposits. Mineral deposits of New Zealand, vol 13. Australasian Institute of Mining and Metallurgy Monograph, pp 89–91

[CR85] Tassi F, Nisi B, Cardellini C, Capecchiacci F, Donnini M, Vaselli O, Avino R, Chiodini G (2013) Diffuse soil emission of hydrothermal gases (CO2, CH4, and C6H6) at Solfatara crater (Campi Flegrei, southern Italy). Appl Geochem 35:142–153

[CR86] Taussi M, Nisi B, Pizarro M, Morata D, Veloso EA, Volpi G, Vaselli O, Renzulli A (2019) Sealing capacity of clay-cap units above the Cerro Pabellón hidden geothermal system (northern Chile) derived by soil CO2 flux and temperature measurements. J Volcanol Geoth Res 384:1–14

[CR87] Umwelt-Geräte-Technik (2014) User manual PL-300. https://ugt-online.de/fileadmin/Public/downloads/Produkte/Bodenkunde/Leitfaehigkeit/PL_300_en_Ver02.pdf:1-18

[CR88] Umwelt-Geräte-Technik (2019) Bedienungsanleitung PL-300. https://manualslib.de/manual/493187/Ugt-Pl-300.html

[CR89] Viveiros F, Ferreira T, Silva C, Gaspar JL (2009) Meteorological factors controlling soil gases and indoor CO2 concentration: a permanent risk in degassing areas. Sci Total Environ 407(4):1362–1372 18996571 10.1016/j.scitotenv.2008.10.009

[CR90] Viveiros F, Cardellini C, Ferreira T, Caliro S, Chiodini G, Silva C (2010) Soil CO2 emissions at Furnas volcano, São Miguel Island, Azores archipelago: volcano monitoring perspectives, geomorphologic studies, and land use planning application. Journal of Geophysical Research: Solid Earth 115(B12)

[CR91] Werner C, Aiuppa A, Edmonds M, Crdellini C, Carn S, Chiodini G, Cottrell E, Burton M, Shinohara H, Allard P (2019) Carbon dioxide emissions from subaerial volcanic regions: two decades in review. Deep Carbon, past to Present 1:188–236

[CR93] Winick J, Powell T, Mroczek E (2011) The natural-state geochemistry of the Rotokawa reservoir. Proc. NZ Geothermal Workshop

[CR94] Yang T-H, Chambefort I, Rowe M, Mazot A, Seward A, Werner C, Fischer T, Seastres J, Siega F, Macdonald N (2024) Variability in surface CO2 flux: implication for monitoring surface emission from geothermal fields. Geothermics 120:102981, 102981

