# Supplementary material for: Shallow permeability structure and gas flow in hydrothermally altered soils at the Rotokawa Geothermal Field, New Zealand
Source: Bull Volcanol. 2026 Jun 23;88(7):73. doi: 10.1007/s00445-026-02002-7 (PMC13287163; doi:10.1007/s00445-026-02002-7)
Supplement: Supplementary file 2 — (DOCX 6.34 MB) [file 445_2026_2002_MOESM2_ESM.docx]

# Supplementary Materials


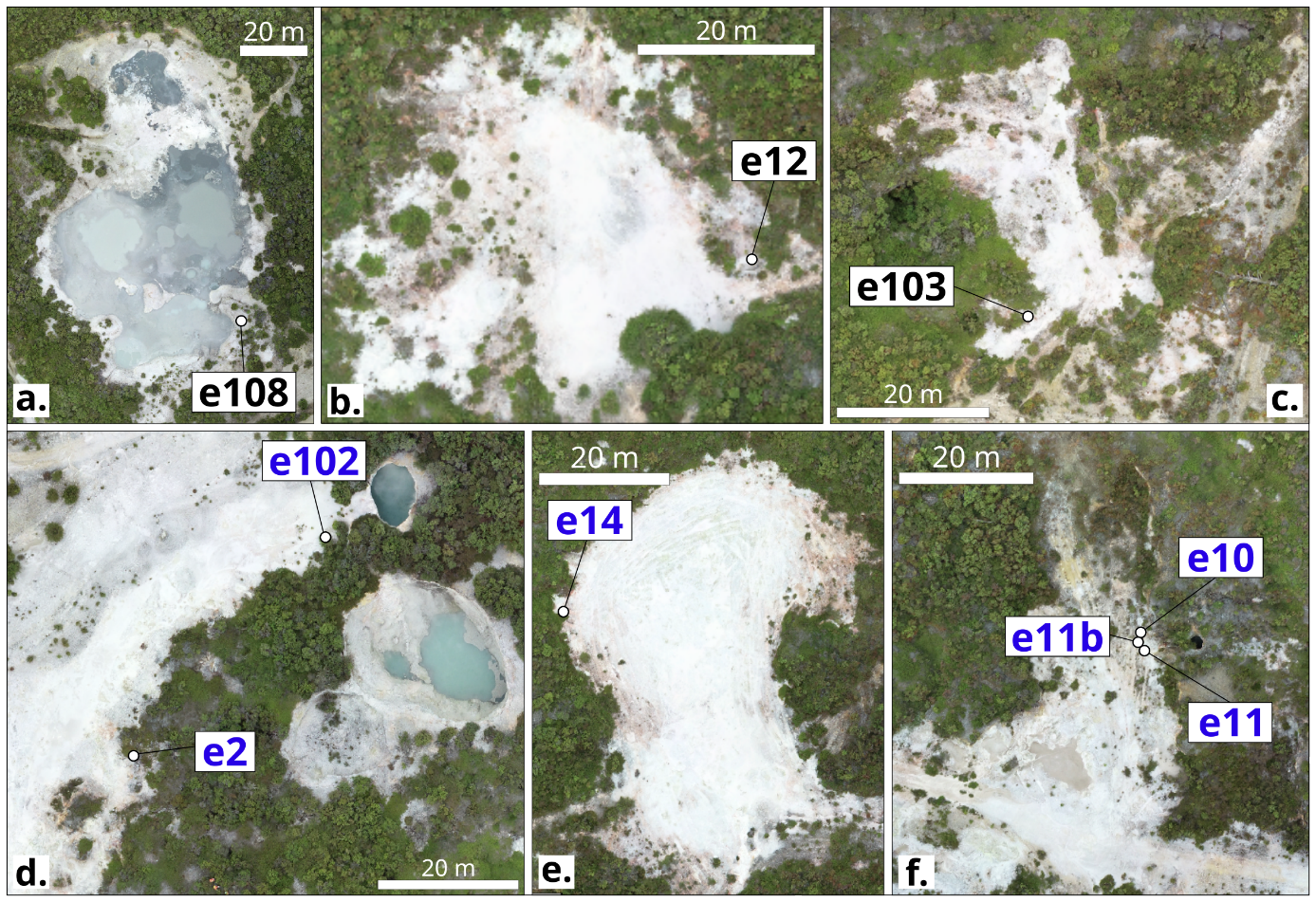


**Supplementary Figure 1** Close up aerial images of sampling sites e108 (**a**), e12 (**b**), e103 (**c**), e2 and e102 (**d**), e14 (**e**), e10, e11 and e11b (**f**), all situated within the ash- and pumice-rich surficial deposits of the Taupō Pumice Formation (in blue the sites where we performed in situ gas and petrophysical properties measurements and in black the ones where we performed only petrophysical properties measurements


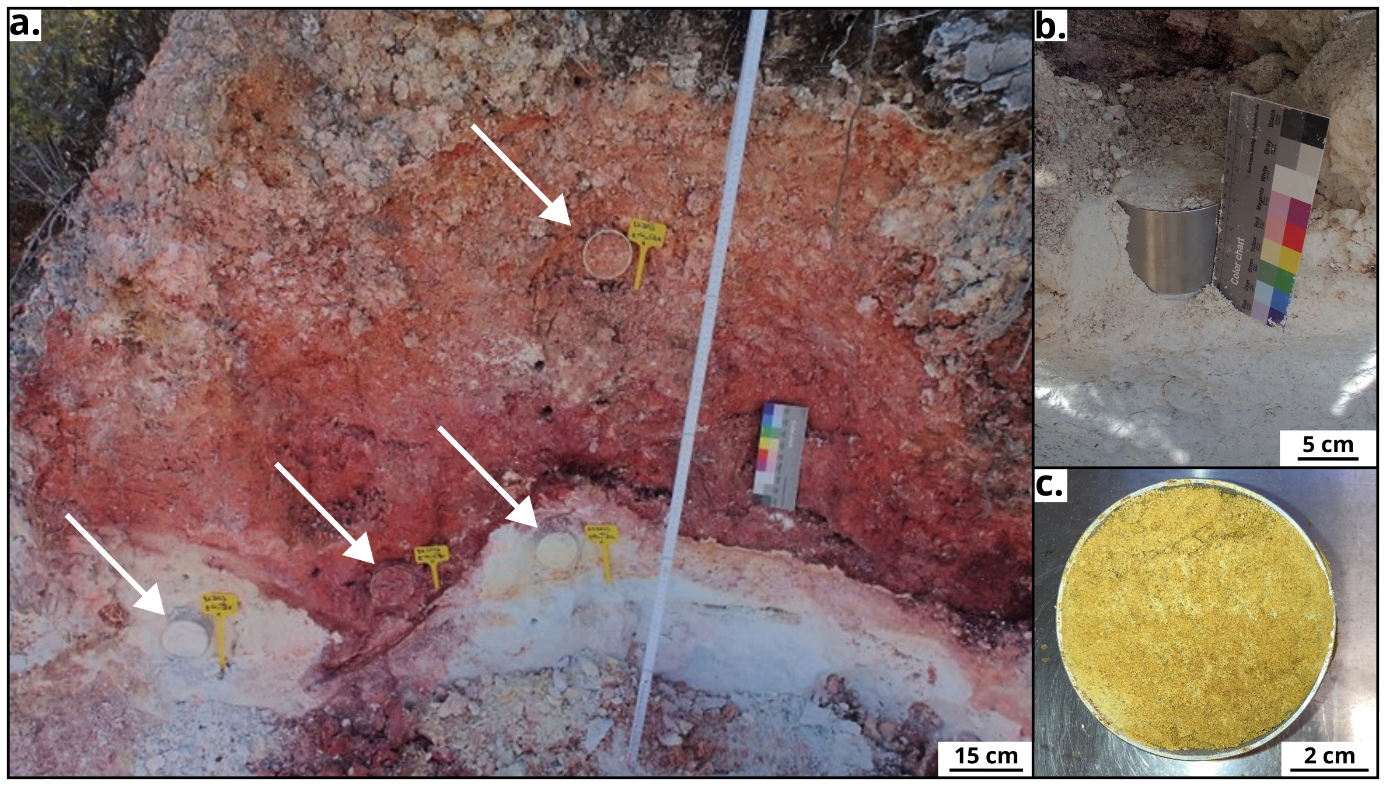


**Supplementary Figure 2** Sampling of soil samples with stainless steel cylinders. **(a)** Cylinders inserted horizontally into the different soil layers (indicated by white arrows); **(b)** close-up image of the retrieval of a cylinder inserted vertically into the soil; **(c)** top view of a soil cylinder a few hours after collection.

| **Supplementary Table S1.** Classification, origin, and key textural characteristics of surficial soil units at Rotokawa. | | | | |
| --- | --- | --- | --- | --- |
| **Soil unit/group** | **Origin** | **Main texture/composition** | **Typical occurrence** | **Notes** |
| *T1–T3* | Taupō Pumice fall deposits | pumice-rich, silt–sand to clay-enriched | undisturbed sequences | increasing alteration from T1/T2 to T3 |
| *T4–T5* | Pyroclastic flow deposits | coarse pumice-rich layers | primary units | T5 generally more altered |
| *E1–E3* | Excavated/reworked mining material | heterogeneous pumice, altered tephra, clay-rich fragments | mined areas | anthropogenic reworking |
| *C1–C2* | Clay-rich reworked horizons | clay-enriched, commonly wet | contacts between primary and reworked units | locally semi-confining |
| *M* | Mud-pool-related deposits | plastic clay-rich soils | excavated depressions | steam condensation/boiling influence |
| *O* | Organic/oxidised soils | silt–sand beds with roots/organic matter | away from active features | less reworked |
| *Br* | Breccia/collapse material | angular coarse debris | collapse/subsidence areas | variable permeability |
